# Variation in CD8 T cell IFNγ differentiation to strains of *Toxoplasma gondii* is characterized by small effect QTLs with contribution from ROP16

**DOI:** 10.3389/fcimb.2023.1130965

**Published:** 2023-05-23

**Authors:** Angel K. Kongsomboonvech, Laura García-López, Ferdinand Njume, Felipe Rodriguez, Scott P. Souza, Alex Rosenberg, Kirk D. C. Jensen

**Affiliations:** ^1^ Department of Molecular and Cell Biology, University of California, Merced, Merced, CA, United States; ^2^ Quantitative Systems Biology Graduate Program, University of California, Merced, Merced, CA, United States; ^3^ The Center for Tropical and Emerging Global Diseases, University of Georgia, Athens, GA, United States; ^4^ Health Sciences Research Institute, University of California, Merced, Merced, CA, United States

**Keywords:** *Toxoplasm gondii*, CD8 T cell, QTL (quantitative trait loci), IFN-gamma, GRA43, TgNSM, RIPK3, ROP16

## Abstract

**Introduction:**

*Toxoplasma gondii* induces a strong CD8 T cell response characterized by the secretion of IFNγ that promotes host survival during infection. The initiation of CD8 T cell IFNγ responses *in vitro* differs widely between clonal lineage strains of *T. gondii*, in which type I strains are low inducers, while types II and III strains are high inducers. We hypothesized this phenotype is due to a polymorphic “Regulator Of CD8 T cell Response” (ROCTR).

**Methods:**

Therefore, we screened F1 progeny from genetic crosses between the clonal lineage strains to identify ROCTR. Naïve antigen-specific CD8 T cells (T57) isolated from transnuclear mice, which are specific for the endogenous and vacuolar TGD057 antigen, were measured for their ability to become activated, transcribe *Ifng* and produce IFNγ in response to *T. gondii* infected macrophages.

**Results:**

Genetic mapping returned four non-interacting quantitative trait loci (QTL) with small effect on *T. gondii* chromosomes (chr) VIIb-VIII, X and XII. These loci encompass multiple gene candidates highlighted by ROP16 (chrVIIb-VIII), GRA35 (chrX), TgNSM (chrX), and a pair of uncharacterized NTPases (chrXII), whose locus we report to be significantly truncated in the type I RH background. Although none of the chromosome X and XII candidates bore evidence for regulating CD8 T cell IFNγ responses, type I variants of ROP16 lowered *Ifng* transcription early after T cell activation. During our search for ROCTR, we also noted the parasitophorous vacuole membrane (PVM) targeting factor for dense granules (GRAs), GRA43, repressed the response suggesting PVM-associated GRAs are important for CD8 T cell activation. Furthermore, RIPK3 expression in macrophages was an absolute requirement for CD8 T cell IFNγ differentiation implicating the necroptosis pathway in T cell immunity to *T. gondii*.

**Discussion:**

Collectively, our data suggest that while CD8 T cell IFNγ production to *T. gondii* strains vary dramatically, it is not controlled by a single polymorphism with strong effect. However, early in the differentiation process, polymorphisms in ROP16 can regulate commitment of responding CD8 T cells to IFNγ production which may have bearing on immunity to *T. gondii*.

## 1 Introduction

*Toxoplasma gondii* is an intracellular pathogen responsible for toxoplasmosis, an underdiagnosed and neglected parasitic disease. *T. gondii* is considered one of the most successful parasites as it can accommodate a wide host range, infecting nearly all warm-blooded vertebrates including an estimated one-third of the world’s human population. Upon invasion, *T. gondii* forms and sequesters itself in a parasitophorous vacuole (PV) that does not initially fuse with host organelles (Sinai et al., 1997; Coppens, 2017), shielding itself from the cytosolic immune sensing mechanisms and defense machinery aimed at its elimination. Host cytotoxic CD8 T cells respond to intracellular pathogens such as *T. gondii* through recognition of cytosolic-derived peptide antigens presented by MHC I molecules on the surface of infected cells (Dzierszinski et al., 2007). If detected, CD8 T cells will secrete the pro-inflammatory cytokine, interferon-gamma (IFNγ) to combat protozoan infections. This CD8 T cell-mediated IFNγ response is required for the elimination of *T. gondii* (Suzuki and Remington, 1990; Gazzinelli et al., 1991; Gazzinelli et al., 1992; Wang et al., 2004; Nishiyama et al., 2020; Suzuki, 2020). In turn, IFNγ activates the Janus kinase-signal transducer and activator of transcription 1 (Jak/STAT1) signaling pathway, inducing the Immunity-Related GTPases (IRGs) (Ling et al., 2006; Hunn et al., 2008; Kim et al., 2012; Haldar et al., 2013). IRGs then bind to the parasite’s PV membrane (PVM) and disrupt it through GTPase-driven IRG oligomerization on the PVM (Hunn et al., 2008). The IRG pathway is necessary for the CD8 T cell response to antigens that are sequestered inside the PV of *T. gondii* (Jensen, 2016), which includes the model antigen OVA engineered to be secreted into the lumen of the PV (Gubbels et al., 2005; Gregg et al., 2011; Lee et al., 2015; Rommereim et al., 2019) and the PV-associated TGD057 antigen (Kongsomboonvech et al., 2020). Inflammasomes, particularly NLRP3 and NLRP1 inflammasome complexes, have also been shown to recognize and control *T. gondii* infections in a variety of species (Witola et al., 2011; Cirelli et al., 2014; Ewald et al., 2014; Gorfu et al., 2014; Gov et al., 2017). We have recently shown that NLRP sensors, but not the inflammasome complexes are required for full induction of the naïve CD8 T cell IFNγ response to parasite-infected cells (Kongsomboonvech et al., 2020).

*T. gondii* utilizes various virulence factors to combat host immune responses to ensure its survival. However, *T. gondii* must achieve a ‘balance’ where it can establish a chronic infection and produce tissue cysts, an infectious form that allows transmission between hosts following oral consumption. This balance may not be achievable in every species, or individual host within a species, for which all warm-blooded animals are believed to be suitable for *T. gondii* infection. This has led to the hypothesis that specific strains of *T. gondii* have adapted to various hosts to achieve balance through use of polymorphic virulence factors, but this balance does not translate to every host (Boothroyd, 2009). For example, *T. gondii* strains differ dramatically in virulence within laboratory mice (Howe and Sibley, 1995) and with the severity of human toxoplasmosis (Grigg et al., 2001; Khan et al., 2006; de-la-Torre et al., 2007; Campos et al., 2008; McLeod et al., 2012; de-la-Torre et al., 2013). Of the clonal lineages endemic to North America and Europe, type I strains are highly virulent. Its lethal dose (LD_100_) is 1 parasite for laboratory mouse infections (Pfefferkorn and Pfefferkorn, 1976), and kills the mice prior to establishing chronic infection. Types II and III are less virulent than type I, with a LD_100_ of approximately 10^3^ and 10^5^, respectively (Sibley and Boothroyd, 1992). The genetically diverse “atypical” strains commonly found in South America are also extremely virulent in mice (Fux et al., 2007; Khan et al., 2011; Jensen et al., 2015), and human ocular toxoplasmosis is more severe in South America compared to other locales (Grigg et al., 2001; Khan et al., 2006; Campos et al., 2008; Sauer et al., 2011). In contrast, type I strains fail to cause disease in Lewis rats (Wang et al., 2019), other subspecies of *Mus musculus* (Lilue et al., 2013) and certain farm animals, including pigs and poultry which are relatively refractory to toxoplasmosis across the globe (Stelzer et al., 2019). Whether *T. gondii* requires unique virulence strategies to infect the various hosts they encounter in nature is unknown. Various species differ with respect to the exact mechanism by which *T. gondii* is killed and immune pathways available for parasitic resistance (Gazzinelli et al., 2014; Saeij and Frickel, 2017; Mukhopadhyay et al., 2020). Hence, manipulation of CD8 T cell responses by *T. gondii* might represent one of a diverse set of strategies needed to achieve balanced infections across a broad host range.

The use of genetic crosses has proven fruitful for discovery of polymorphic virulence factors that intersect host-parasite interactions relevant to *T. gondii* and its murine host. The F1 progeny of the clonal lineages (from type I x type II, type I x type III, and type II x type III sexual crosses) differ greatly in virulence phenotypes (Saeij et al., 2006; Taylor et al., 2006; Behnke et al., 2011; Reese et al., 2011). Through quantitative trait loci (QTL) mapping using these F1 progeny, several *T. gondii* proteins contributing to virulence have been identified, namely three polymorphic rhoptry proteins (ROPs): ROP16 (Saeij et al., 2006; Saeij et al., 2007), ROP18 (Saeij et al., 2006; Taylor et al., 2006), and a family of ROP5 pseudokinases (Behnke et al., 2011; Reese et al., 2011). Polymorphisms in the tyrosine kinase ROP16, expressed in type I and type III (ROP16_I/III_) but not type II (ROP16_II_) strains, allow sustained phosphorylation of STAT3, STAT5 and STAT6, inducing alternative activation (M2) of macrophages and dampening of host IL-12 production (Yamamoto et al., 2009; Ong et al., 2010; Butcher et al., 2011; Jensen et al., 2011). In the type III genetic background, ROP16_III_ promotes virulence due to early M2 activation and subsequent suppression of Th1 immunity and CD8 T cell responses (Tuladhar et al., 2019; Chen et al., 2020). In contrast, when ROP16_I_ or ROP16_III_ are expressed as a transgene in the type II background, it promotes host resistance (Saeij et al., 2006; Jensen et al., 2011) and parasite killing by a mechanism that is dependent on the genetic background of the parasite (Jensen et al., 2013). The serine threonine kinase ROP18 phosphorylates and inactivates IRGs, protecting the PV from destruction (Fentress et al., 2010; Steinfeldt et al., 2010; Fleckenstein et al., 2012; Niedelman et al., 2012). However, ROP18 is not expressed in type III *T. gondii* strains (Saeij et al., 2006; Taylor et al., 2006), explaining its lesser degree of virulence compared to types I and II *T. gondii* strains. The pseudokinase ROP5 binds to IRGs, preventing them from oligomerizing and accumulating at the PV (Hunn et al., 2008; Howard et al., 2011; Reese et al., 2014). In contrast, the ROP5 variants expressed by type II *T. gondii* strains fail to perform this function rendering type II strains less virulent (Niedelman et al., 2012; Reese et al., 2014). F1 progeny from a cross between the highly virulent atypical strain VAND and a type II strain further demonstrate the importance of ROP5 polymorphisms in regulating parasite virulence (Behnke et al., 2015). Genetic mapping using F1 progeny have been instrumental for discovery of parasite effectors that mediate specific host processes, including the identification of host mitochondrial-PV association factor MAF1 (Pernas et al., 2014) and secreted NTPases that limit host cellular concentrations of NTP (Olias and Sibley, 2016). Hence, QTL mapping has proven robust for the identification of effectors that mediate a range of strain-specific phenotypes of *T. gondii*.

Recently we reported strain-specific differences in host CD8 T cell IFNγ responses to *T. gondii* infections (Kongsomboonvech et al., 2020). Type I strains and other clade A isolates induced relatively low amounts of IFNγ secretion from naïve CD8 T cells, but most other strains induced relatively high CD8 T cell IFNγ responses, a phenotypic pattern that does not correlate with polymorphisms of known parasite effectors (Kongsomboonvech et al., 2020). Here, we report our attempts to identify *T. gondii* Regulator(s) Of CD8 T cell Responses, or “ROCTRs”, that are responsible for strain-differences in eliciting CD8 T cell IFNγ responses. We utilized an experimental approach by which naïve antigen-specific CD8 T cells that bear specificity to a conserved *T. gondii* endogenous antigen, TGD057, were analyzed for IFNγ responses to parasite-infected bone marrow-derived macrophages (BMDMs). QTL analysis of the F1 progeny from the type I x type II cross suggested ROCTRs are encoded on *T. gondii* chromosomes X and XII with very weak effect, while our interrogation of a significant additive-QTL on chromosome VIIb-VIII, revealed that ROP16 regulates the early IFNγ transcriptional response of activated CD8 T cells. Finally, we present evidence that the host’s necroptosis pathway and *T. gondii* effectors known to mediate delivery of GRAs to the PVM appear to regulate CD8 T cell IFNγ differentiation.

## 2 Materials and methods

### 2.1 Parasite strains and passaging

*Toxoplasma gondii* strains were serially passaged in ‘Toxo medium’ [4.5 g/liter D-glucose in DMEM with GlutaMAX (Gibco, cat# 10566024), 1% heat-inactivated fetal bovine serum (FBS) (Omega Scientific, cat# FB-11, lot# 441164), 1% penicillin-streptomycin (Gibco, cat# 15140122)], in confluent flasks of monolayers of human foreskin fibroblasts (HFFs) and cultured at 37°C, 5% CO_2_. HFFs were cultured in ‘HFF medium’ [4.5 g/liter D-glucose in DMEM with GlutaMAX (Gibco), 20% heat-inactivated FBS (Omega Scientific), 1% penicillin-streptomycin (Gibco), 0.2% Gentamicin (Gibco, cat# 15710072), 1X L-Glutamine (Gibco, cat# 21051024)]. Strains assayed are listed in **Table S1**, some are generous gifts from Jeroen Saeij (University of California, Davis) and David Sibley (Washington University, St. Louis), and others were obtained from BEI Resources.

### 2.2 Mice and generation of bone marrow-derived macrophages

Transnuclear T57 (Kirak et al., 2010) and T-GREAT mice were bred in-house under specific pathogen free (SPF) conditions. T-GREAT mice (Kongsomboonvech et al., 2020) bear the same T cell receptor specificity as T57, but also express an *Ifng :* YFP reporter that allows measurement of *Ifng* transcript by detection of YFP fluorescence (Reinhardt et al., 2015). C57BL/6J (B6) (colony 000664) and *P2x7r*-/- (colony 005576) mice were purchased from Jackson Laboratories and kept in-house under SPF conditions. Hind bones from *Ripk3*-/- mice, B6.129-*Ripk3^tm1Vmd^* (Newton et al., 2004), were provided by Laura Knoll (University of Wisconsin). Bone marrow cells from the hind bones of 6-8 weeks old mice were obtained and cultured in ‘BMDM medium’ [4.5 g/liter D-glucose in DMEM with GlutaMAX (Gibco), 20% heat-inactivated FBS (Omega Scientific), 1% penicillin-streptomycin (Gibco), 1X non-essential amino acids (Gibco, cat# 11140076), 1 mM sodium pyruvate (Gibco, cat# 11360070)] supplemented with 20% L929 conditioned medium, and then harvested after 6-7 days of differentiation. All animal protocols were approved by UC Merced’s Committee on Institutional Animal Care and Use Committee (AUP 20-0015). All mouse work was performed in accordance to the *Guide to the Care and Use of Laboratory Animals* of the National Institutes of Health and the Animal Welfare Act (assurance number A4561-1). Euthanasia of mice was performed by inhalation of CO_2_ to effect of 1.8 liters per minute.

### 2.3 Measuring the naïve T57 CD8 T cell IFNγ response to parasite-infected BMDMs

B6 BMDMs were plated at 2x10^5^ cells per well in a 96-well tissue culture-treated plate, in BMDM medium supplemented with 10% L929 conditioned medium, and incubated overnight. The next day, BMDMs were infected with *T. gondii* tachyzoites, in triplicates, in ‘T cell medium’ [RPMI 1640 with GlutaMAX (Gibco, cat# 61870127), 20% heat-inactivated FBS (Omega Scientific, cat# FB-11, lot# 441164), 1% penicillin-streptomycin (Gibco, cat#15140122), 1 mM sodium pyruvate (Gibco, cat# 11360070), 10 mM HEPES (Gibco), 1.75 µl of β-mercaptoethanol (Gibco, cat# 21985023) per 500 mL RPMI 1640 with GlutaMAX]. The infections were done at multiplicity of infection (MOI) of 0.6, 0.2, and 0.07. Approximately 2 hours post-infection, 5x10^5^ naïve lymph node cells and splenocytes obtained from a naïve T57 transnuclear mouse were added into all wells of infected BMDMs. The lymph nodes and spleens were processed and combined, and red blood cells were lysed with ammonium chloride-potassium (ACK) lysis buffer, prior to being added to the co-culture. The supernatants of the co-cultures were harvested 48 hours later for further analysis by ELISA according to the manufacturer’s instructions (Invitrogen eBioscience, cat# 88731477). The supernatants were analyzed at 1:2, 1:20, and 1:200 dilutions to obtain values within the linear range of the manufacture’s ELISA standards.

### 2.4 Measuring *Ifng* transcription by flow cytometry of T-GREAT cells

T-GREAT cells were co-cultured with infected BMDMs as described above for T57 CD8 T cells. After 14 to 18 hours of the co-culture, cells were harvested for flow cytometry. With preparations all done on ice, cells were washed with ‘FACS buffer’ [PBS pH 7.4 (Gibco, cat# 10010049), 2% heat-inactivated FBS (Omega Scientific)] and blocked with ‘blocking buffer’ [FACS buffer with 5% normal Syrian hamster serum (Jackson Immunoresearch, cat# 007-000-120), 5% normal mouse serum (Jackson Immunoresearch, cat# 015-000-120), and anti-mouse CD16/CD32 FcBlock (BD Biosciences, clone 2.4G2) at 1:100 dilution)]. Samples were stained at 1:120 dilution with fluorophore-conjugated anti-mouse monoclonal antibodies against CD8α PE (eBioscience) or BV510 (BioLegend) (clone 53–6.7), CD3ϵ APC-eFlour780 (eBioscience, clone 17A2), CD62L eFlour450 (eBioscience, clone MEL-14), and CD69 APC (BioLegend, clone H1.2F3). Samples were washed and then incubated with propidium iodide (PI) at 1:1000 dilution (Sigma, cat# P4170). Flow cytometry was performed on an LSRII (Becton Dickinson) or ZE5 (Bio-Rad) analyzer and processed with FlowJo software (version 10.8.1); PI+ cells were excluded from analysis.

### 2.5 Correction for relative viability between parasite strains

HFFs were plated in 24-well tissue culture-treated plates in HFF medium. Confluent monolayer HFFs were infected with 100 and 300 parasites. Plaques were quantified 4-6 days after infection. Displayed results are from MOIs with similar viability, the equivalent of ~MOI 0.2 was chosen for most assays.

### 2.6 Genetic linkage analysis and Quantitative Trait Loci (QTL) mapping

The QTL analysis for the CD8 T cell IFNγ response phenotype, including both 1D (scanone) and 2D scans (scantwo) as well as the effect plots, was performed using ‘R’ version 3.6.1 and the ‘R/qtl’ package (Broman et al., 2003). The concentration of CD8 T cell-secreted IFNγ that was obtained for each F1 IxII progeny was normalized to that of type II strain, and for the F1 IxIII progeny, the values were normalized to the type III strain. The average of normalized values obtained over multiple experiments was then Log10 transformed and used for the QTL analysis. For the F1 IIxIII progeny, the Log10 of the CD8 T cell IFNγ concentration from a single experiment was used. A thousand permutations were calculated to obtain significant threshold values (p ≤ 0.05 and p ≤ 0.1). Genetic markers and the allelic assignments for each F1 progeny were obtained from previous studies (Su et al., 2002; Saeij et al., 2006; Behnke et al., 2011) and publicly available databases (http://toxomap.wustl.edu/IxIII_Typing_Table.html). The genetic maps of chromosomes VIIb and VIII were stitched into a single chromosome and renamed as “VIIb-VIII”. Recent evidence suggests these are a single chromosome (Bunnik et al., 2019; Xia et al., 2021), and our revised genetic map reflects the correct genetic marker orientation in the stitched VIIb-VIII *T. gondii* chromosome.

### 2.7 Generation of gene knockout parasite strains

To generate a double knockout strains, Cas9-expressing pSS013 plasmid (gift from Jeroen Saeij, University of California, Davis) containing single guide RNAs (sgRNAs) targeting exon 1 of the candidate ROCTR genes, TG_278878 and TG_278882, were co-transfected *via* electroporation with selectable markers hypoxanthine-guanine phosphoribosyl transferase (*HXGPRT*) or dihydrofolate reductase (*DHFR-TS*) into RH *Δhxgprt Δku80* or ME49 *Δhxgprt::FLUC* (ME49 *Δhpt*), respectively, at a 5:1 ratio of sgRNAs/plasmid to PCR amplicon of the selectable marker. The *HXGPRT* amplicon was generated using the pTKO-att plasmid as the template DNA and the *DHFR-TS* amplicon was generated using the pLoxP-DHFR-mCherry plasmid as the template DNA. In the case of the HXGPRT amplicon, 20 bp of homology arms were introduced during the PCR to allow for homology directed repair and removal of the TG_278878 and TG_278882 in the RH *Δhxgprt Δku80* genetic background. The transfection bulk populations were then selected with ‘MPA xanthine’ medium [4.5 g/liter D-glucose in DMEM with GlutaMAX (Gibco, cat# 10566024), 1% heat-inactivated fetal bovine serum (FBS) (Omega Scientific, cat# FB-11, lot# 441164), 1% penicillin-streptomycin (Gibco, cat# 15140122), 0.05 mg/ml mycophenolic acid (MPA) (Millipore Sigma, cat# 475913), 0.05 mg/ml xanthine (Alfa Aesar; cat# AAA11077-22)] or in Toxo medium containing 3 µM pyrimethamine (Millipore Sigma, cat# 46706) to select for the presence of *HXGPRT* or *DHFR-TS* insertion, respectively. The drug selected population was screened by diagnostic PCR for evidence of successful targeting and repair of the Cas9 cut site with the selectable marker and then cloned by limiting dilution in a 96-well plate in MPA xanthine or pyrimethamine selection medium. Wells with only one clone were isolated and candidate gene disruption was again confirmed by diagnostic PCR for successful marker integration and double gene deletion. Parasite genomic DNA was isolated using DNAzol (Invitrogen, cat# 10503027) and ethanol precipitation. Diagnostic PCR was performed with MangoMix, which contains MangoTaq DNA polymerase (Bioline, cat# BIO-25-33). PCR amplicons of the selectable markers were generated with Phusion polymerase (NEB, cat# M0530L). Sequencing of PCR products and plasmids was performed by UC Berkeley DNA Sequencing Facility and samples were prepared according to their protocol. Sequencing results were analyzed using Sequencher 5.4.6 and SnapGene Viewer 5.2.1. All oligos and plasmids used in this study can be found in **Table S2**. The RH *Δnsm*, RH *Δist* and RH *Δist Δnsm* strains were made in an analogous manner as that previously described for ME49 (Rosenberg and Sibley, 2021).

### 2.8 Immunofluorescence assay

HFFs were plated on coverslips with HFF medium in 24-well tissue culture-treated plates. Once confluent, the monolayer HFFs were infected with *T. gondii* and incubated at 37°C, 5% CO_2_ for 16 hours. The samples were then fixed with 3% formaldehyde in phosphate buffered saline (PBS) for 20 minutes. After the fixation, these were blocked with blocking buffer (3% BSA, 5% normal goat serum, 0.2% Triton X-100, 0.1% sodium azide in PBS). To visualize GRA5, the infected cells were stained with mouse anti-GRA5 primary monoclonal antibody (BioVision, clone TG 17.113) at 1:500 dilution, followed by Alexa Fluor 488 goat anti-mouse IgG (Life Technologies, cat # A11029) secondary antibody at 1:3000 dilution. To visualize TGD057, the infected samples were stained with rabbit anti-TGD057 polyclonal antibody (gift from Nicolas Blanchard, INSERM) at 1:2000 dilution, followed by Alexa Fluor 594 goat anti-rabbit IgG (Life Technologies, cat# A11037) secondary antibody at 1:3000 dilution.

### 2.9 Western analysis

*T. gondii* lysates were prepared through syringe-lysis, resuspended in Laemmli buffer (125 mM Tris HCl, 30% glycerol, 2% SDS, 0.2% bromophenol blue), and denatured at 90-100°C for 5 minutes. The lysates were separated on 4-20% Mini-PROTEAN TGX pre-cast 540 gels (Bio-Rad, cat# 4561096) or 5-15% polyacrylamide gels and then transferred to nitrocellulose membranes. The membranes were blocked with 10% fortified bovine milk in ‘TBS-T 0.1%’ [Tris-Buffered Saline and 0.1% Tween] at room temperature for 1h, and then incubated with α-TGD057 antibody (gift from Nicolas Blanchard, INSERM) at 1:4000 dilution in TBS-T 0.1% overnight at 4°C. Membranes were washed with TBS-T 0.1% three times and later incubated with goat α-rabbit horseradish peroxidase (HRP)-conjugated antibodies (Southern Biotech, cat# 4030-05) at 1:4000 dilution for 1h at room temperature. Membranes were then washed again TBS-T 0.1% three times and developed with Immobilon^®^ Forte Western HRP Substrate (Millipore, cat# WBLUF0500). All blots were imaged *via* chemiluminescence on a ChemiDoc Touch (Bio-Rad, cat# 12003153).

### 2.10 Statistical analysis and normalization between experiments

Bar graphs represent the average value obtained for all experiments, with standard deviations indicated. For these, values from individual experiments are represented as dots. For each measured experiment, results between parasites strains or conditions were mainly represented as that relative to the T57 response elicited by wildtype BMDMs infected with type II strains (equal to 1). For data with normal distribution, one-way or two-way ANOVA with Bonferroni’s *ad-hoc* statistical tests were determined. For data with non-Gaussian distribution, Kruskal-Wallis and Dunn’s *ad-hoc* test were applied. P-values < 0.05 were considered significant. Statistical analyses were performed with GraphPad Prism version 8.3.0.

## 3 Results

### 3.1 ROCTRs with small effect are encoded on *Toxoplasma gondii* chromosomes X and XII

**Figure 1:**
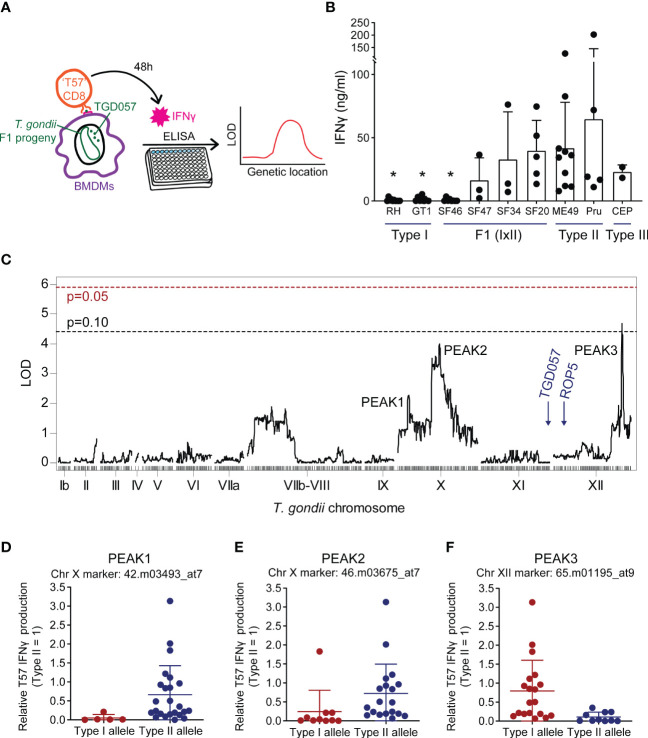
Naïve TGD057-specific CD8 T cell IFNγ responses to *Toxoplasma gondii* are potentially modulated by genetic loci on chromosomes X and XII. **(A)** Schematic of the strategy to identify “ROCTR”. TGD057-specific CD8 T cell IFNγ responses to *T. gondii*-infected bone marrow-derived macrophages (BMDMs) were measured. Two hours post-infection, naïve antigen-specific TGD057 (‘T57’) CD8 T cells obtained from transnuclear mice were added to the infected BMDMs. Supernatant from the co-culture was harvested 48h later and then analyzed for IFNγ concentration by ELISA. Genetic linkage analyses were performed with IFNγ response values obtained from the F1 progeny of crosses between the clonal lineage strains to find quantitative trait loci responsible for the phenotype. **(B)** T57 IFNγ responses to the indicated clonal lineage strains and selected F1 progeny from the IxII genetic cross are indicated, average of 2-5 experiments +SD (standard deviation) is plotted; each dot represents the result from an individual experiment. Statistical analysis was performed using one-way ANOVA with Bonferroni’s correction comparing to Pru; *p ≤ 0.05. **(C)** Genetic linkage analysis of T57 IFNγ responses to F1 progeny of the IxII genetic cross were analyzed. The running LOD score for each genetic marker is plotted. The QTL map reveals regions of interest on *T. gondii* chromosomes X and XII. QTLs with LOD scores > 2 are labeled as PEAK1, PEAK2, and PEAK3. The LOD significant threshold values are calculated following 1,000 permutations, indicated with dashed lines and p-values (p = 0.05 in red, p = 0.10 in black). The genetic locations of *ROP5* and the *TGD057* antigen are shown for reference. **(D−F)** Effect plots for the genetic markers corresponding to PEAKS 1 through 3, are shown. The IFNγ responses are normalized to that induced by the wildtype (WT) type II strain. Each dot represents the average value obtained for each F1 IxII strain.

To identify *T. gondii* genetic loci responsible for the strain-differences in CD8 T cell responses to infections, and ultimately ROCTR, we assessed 29 strains of F1 progeny from a type I x type II cross (F1 IxII), 34 strains of F1 progeny from a type II x type III cross (F1 IIxIII), and 32 strains of F1 progeny from a type I x type III cross (F1 IxIII) in an antigen-specific CD8 T cell activation assay (**Figure 1A**) (Kongsomboonvech et al., 2020). In brief, C57BL/6J bone marrow-derived macrophages (BMDMs) were infected with *T. gondii* and co-cultured with splenocytes and lymph node cells from naïve transnuclear ‘T57’ mice. These mice were cloned from the nucleus of a single tetramer-positive *T. gondii*-specific CD8 T cell which have a single T cell receptor specific for the TGD057_96-103_ epitope (T57 epitope) presented by H-2K^b^ MHC I (Kirak et al., 2010; Wilson et al., 2010). The supernatant was harvested at 48 hours of the co-culture and analyzed for IFNγ concentration by ELISA. Although the TGD057_96-103_ epitope is conserved among all *T. gondii* strains, type I parasite strains (RH, GT1) induce low T57-specific CD8 T cell-mediated IFNγ responses, while type II (ME49, Pru) and type III strains (CEP) induce a much higher response (**Figure 1B**), as previously described (Kongsomboonvech et al., 2020). The CD8 T cell response differs greatly in response to infections of F1 IxII *T. gondii* strains (**Figure S1**). For example, the CD8 T cell IFNγ response to SF46 is quite low, unlike the high IFNγ level elicited in response to SF20 infections (**Figure 1B**). These results suggest the potential to genetically map loci responsible for strain-differences in T57 IFNγ responses to an endogenous antigen, which localizes to the PV (Lopez et al., 2015; Kongsomboonvech et al., 2020) and actin cytoskeleton of the parasite (Wilson et al., 2010).

A genome-wide QTL scan was performed to detect genotype-phenotype correlations using F1 progeny derived from all three sexual crosses of the clonal lineages for the naïve T57 IFNγ response at 48 hours. QTL analysis of the F1 IxII cross revealed three suggestive peaks with an LOD value greater than 2 on *T. gondii* chromosomes X (chrX) and XII (chrXII) (**Figure 1C**). Following genome wide permutation testing (n=1000), the QTL on chromosome XII (“PEAK3”) with the logarithm of odds (LOD) score of 4.4, surpassed a threshold value of p = 0.10, but none of the other QTLs (“PEAK1”, “PEAK2”) returned significant values. Polymorphic candidate genes within the 1.5 LOD interval of PEAK3, a 106 Kb region spanning 13 genes (TGME49_chrXII:5754579-5860469), include most notably two tandem uncharacterized NTPases, TG_278878 and TG_278882 (ToxoDB.org) (**Table 1**). The genetic marker corresponding to the maximal LOD score within PEAK3 (65.m01195_at9) is a SNP adjacent to TG_278878. On chromosome X, there are two noticeable peaks. The major peak towards the right end of chromosome X (PEAK2) is a large 1.1 Mb locus (TGME49_chrX:3782766-4930776) encoding 147 genes and includes the genetic marker 46.m03675_at7, which produced the highest LOD score of 3.6 within this chromosome (**Figure 1C**). The minor peak towards the left end of chromosome X (PEAK1) corresponds to a ~419 Kb region of 48 genes (TGME49_chrX:1383691-1802907) and encompasses the genetic marker 42.m03493_at7 with a LOD score of 2.3 (**Figure 1C**); this marker defines a SNP within the *T. gondii* dense granule GRA35. Notably, the *ROP5* locus, which is a known virulence determinant encoded on chromosome XII and identified using this panel of F1 IxII progeny (Behnke et al., 2011), did not produce an identifiable QTL in our screen. Thus, while ROP5 can inhibit the CD8 T cell response (Rommereim et al., 2019; Kongsomboonvech et al., 2020), polymorphisms in ROP5 do not account for strain-specific differences observed for this phenotype, as previously suggested (Kongsomboonvech et al., 2020). The TGD057 antigen encoded by TG_215980 is similarly expressed and entirely conserved between clonal strains, and no phenotype-genotype correlation was observed at this locus (**Figure 1C**). Based on the effect plots, F1 IxII progeny that express type II alleles at the chromosome X genetic markers 42.m03493_at7 and 46.m03675_at7 induce higher CD8 T cell-mediated IFNγ responses compared to those that express type I alleles at these loci, respectively (**Figures 1D, E**). In contrast, F1 IxII progeny that express a type I allele at the chromosome XII genetic marker 65.m01195_at9 induce higher CD8 T cell responses compared to those that express the type II allele (**Figure 1F**), suggesting the genetic background of type I strains may mask the effect of the putative chrXII ROCTR. In search of loci that potentially modify the function of the ROCTR and lower the detection of significant QTLs, epistatic interactions and ‘interactive’ QTLs were calculated, but none were detected between PEAKS 1, 2, and 3 or other loci within the F1 IxII genetic cross (**Figure S2A**, not shown).

**Table 1:** ROCTR candidates on *Toxoplasma gondii* chromosomes VIIb-VIII, X and XII.

Gene ID	Coordinates	Product description	NonSyn/Syn SNP ratio all strains	Amino acid differences (n) type I vs. type II strains	Average expression in RH/GT1	Average expression in Pru/ME49	Exon number	Predicted signal peptide	Predicted transmembrane domain	Fitness score
PEAK1: Chromosome X - minor peak										
TGME49_226470	TGME49_chrX: 1,607,575 - 1,618,478 (+)	hypothetical protein	2.42	55	10.0	21.4	2	N	N	1.25
TGME49_226390	TGME49_chrX: 1,665,302 - 1,670,080 (+)	hypothetical protein	2.92	17	5.0	5.2	1	N	N	0.04
TGME49_226380*	TGME49_chrX: 1,674,639 - 1,678,229 (+)	GRA35	8.67	11	86.4	135.2	1	Y	Y	1.98
PEAK2: Chromosome X - major peak										
TGME49_223485	TGME49_chrX: 3,784,432 - 3,788,334 (+)	hypothetical protein	10.0	155	46.4	45.0	3	N	N	-1.49
TGME49_223430	TGME49_chrX: 3,850,738 - 3,854,319 (-)	hypothetical protein with putative oxidoreductase activity	0.84	2	5.0	13.3	2	Y	Y	0.66
TGME49_212280	TGME49_chrX: 3,942,555 - 3,944,845 (+)	hypothetical protein	1.5	1	47.8	21.4	1	Y	N	0.69
TGME49_212140	TGME49_chrX: 4,023,907 - 4,035,947 (+)	hypothetical protein	1.01	72	7.0	7.6	8	Y	N	-0.1
TGME49_234230	TGME49_chrX: 4,207,706 - 4,228,971 (-)	hypothetical protein	1.07	40	5.0	10.2	6	N	N	-3.19
TGME49_234270	TGME49_chrX: 4,254,283 - 4,268,067 (+)	hypothetical protein, localizes to the apical complex	1.32	223	16.5	40.6	15	N	N	-0.44
TGME49_234300	TGME49_chrX: 4,285,787 - 4,297,019 (-)	hypothetical protein	2.01	138	5.0	12.9	1	N	N	-0.33
TGME49_234350	TGME49_chrX: 4,305,901 - 4,308,402 (-)	hypothetical protein	1.44	14	10.6	22.6	1	Y	N	-0.73
TGME49_234590*	TGME49_chrX: 4,485,141 - 4,486,247 (-)	hypothetical protein	6.0	24	5.0	5.0	1	N	N	-0.14
**TGME49_235140**	TGME49_chrX: 4,633,596 - 4,638,849 (+)	TgNSM	6.7	52	46.4	52.2	7	N	N	1.41
PEAK3: Chromosome XII										
TGME49_278930	TGME49_chrXII: 5,750,428 - 5,757,826 (+)	Tubulin-tyrosine ligase family protein	0.53	2	23.6	10.0	16	N	N	0.5
**TGME49_278882**	TGME49_chrXII: 5,785,062 - 5,788,158 (-)	GDA1/CD39 (nucleoside phosphatase) family protein	0.52	0 (GT1), 400 truncation (RH)	6.3	5.0	1	Y	Y	0.71
**TGME49_278878**	TGME49_chrXII: 5,790,682 - 5,793,202 (-)	GDA1/CD39 (nucleoside phosphatase) family protein	2.47	1 (GT1), 113 truncation (RH)	7.4	8.3	4	Y	N	0.6
TGME49_278870*	TGME49_chrXII: 5,793,484 - 5,811,106 (+)	Myosin F	0.21	3	44.1	39.8	19	N	N	-3.55
TGME49_278840	TGME49_chrXII: 5,828,465 - 5,832,264 (+)	hypothetical protein	1.53	6	5.0	11.8	3	N	N	1.39
TGME49_278815	TGME49_chrXII: 5,852,666 - 5,861,034 (+)	Putative F-box protein	1.63	71	9.6	19.1	5	N	N	-1.59
PEAK4: Chromosome VIIb-VIII (covariate)										
**TGME49_262730**	TGME49_chrVIIb: 1,053,320 - 1,056,333 (-)	ROP16	2.37	39	284.2	473.1	1	Y	N	1.11
TGME49_262500	TGME49_chrVIIb: 1,208,630 - 1,209,634 (-)	hypothetical protein	4.0	5	353.2	713.7	1	N	N	0.69
TGME49_262400	TGME49_chrVIIb: 1,290,906 - 1,299,909 (-)	Lipase	1.61	46	93.3	124.3	9	N	Y	1.24
TGME49_262050	TGME49_chrVIIb: 1,406,941 - 1,409,624 (+)	ROP39	3.92	29	452.3	526.1	1	N	N	1.97
TGME49_261740	TGME49_chrVIIb: 1,552,958 - 1,554,576 (-)	hypothetical protein (Rhoptry)	5.0	11	3710.4	4082.9	1	N	Y	0.31
TGME49_260800	TGME49_chrVIIb: 2,041,739 - 2,048,418 (+)	hypothetical protein (Dense granule)	2.34	33	15.2	23.3	3	N	N	-5.17
TGME49_260520	TGME49_chrVIIb: 2,190,204 - 2,193,134 (-)	hypothetical protein (Dense granule)	1.9	7	70.1	124.6	2	N	N	1.9
TGME49_260480	TGME49_chrVIIb: 2,216,446 - 2,231,203 (+)	leucine rich repeat-containing protein	1.57	112	5	30.4	21	N	N	-0.9

Top ROCTR candidates within the boundaries of each QTL peak (PEAK1, PEAK2, PEAK3 and PEAK4) are listed with genetic coordinates. The information regarding each gene was obtained from ToxoDB.org. The list includes ROCTR candidates that exhibited a high degree of amino acid polymorphisms between all strains deposited in ToxoDB, and/or number of amino acid substitutions or gene expression differences between type I and II strains; unless indicated the numbers represent amino acid differences between GT1 and ME49 strains. Genes with single exons/and or predicted to have a signal peptide were favored for inclusion as a ROCTR candidate. The average expression of RH and GT1 (type I strains) as well as the average expression of Pru and ME49 (type II strains) were calculated from values provided by Minot et al., 2012. The fitness score was obtained from Sidik et al., 2016. Genes investigated by CRISPR inactivation in this study are in bold. Genes encoding the genetic marker that produced the highest LOD score at each QTL peak are denoted with an asterisk. The genes and genetic markers within the PEAK4 QTL returned the same LOD score.

Other sexual crosses examined included the F1 progeny derived from the IxIII and IIxIII crosses, but QTL mapping revealed no statistically significant peaks (**Figures S3**, **S4**). It can be concluded that the polymorphic rhoptries responsible for strain-differences in virulence previously identified through QTL analyses of these same panels of F1 progeny, such as ROP18 and ROP5 (from both F1 IIxIII and F1 IxIII crosses) encoded on chromosomes VIIa and XII, respectively (Saeij et al., 2006; Taylor et al., 2006; Reese et al., 2011), do not seem to account for the strain-differences in host CD8 T cell IFNγ responses to *T. gondii* infections. Moreover, the small effect QTLs suggest that the T57 CD8 IFNγ response may be controlled by multiple gene loci of *T. gondii* and subject to environmental input during the 48 hours co-culture of the CD8 T cells and infected macrophages. Top polymorphic ROCTR candidates for PEAKS 1, 2 and 3 are included in **Table 1**.

### 3.2 Interrogation of NTPase ROCTR candidates on chromosome XII

Candidate genes on *T. gondii* chromosome XII that correspond to PEAK3 include TG_278878 and its adjacent gene TG_278882, which are nucleoside triphosphate hydrolases (NTPases) of the GDA1/CD39 family of ecto-ATPases with apyrase activity (ToxoDB.org). In general, NTPases are secreted following invasion and localize to the PV lumen and PVM (Bermudes et al., 1994; Sibley et al., 1994). The related NTPases, NTPase I and NTPase II (Bermudes et al., 1994), do not contribute to type I strain virulence in mice but deplete cellular ATP (Olias and Sibley, 2016) and are thought to be important for tachyzoite replication (Nakaar et al., 1999; Asai et al., 2002) and possibly egress (Stommel et al., 1997). Large quantities of parasite-derived NTPase can be detected in the serums of infected mice (Asai et al., 1987) and represents up to 2-3% of the entire protein in the tachyzoite (Asai et al., 1983). NTPases may impact the host response in another way. It is possible that the ATP hydrolysis activity of NTPases may dampen host inflammasome activation in response to *T. gondii* infections (Melo et al., 2011). Inflammasome activation, for both NLRP1 and NLRP3, has been shown to be important for *T. gondii* control (Cirelli et al., 2014; Ewald et al., 2014; Gorfu et al., 2014) and can be triggered through the binding of exogenous ATP to the purinergic receptor P2X7 (Jo et al., 2016; Amores-Iniesta et al., 2017). It is possible that *T. gondii* NTPases deplete the amount of cytosolic ATP, thus preventing inflammasome activation either by lowering available exogenous ATP required for P2X7 receptor (P2X7R) activation following egress, or by thwarting NLR-oligomerization which is an ATP-dependent process (Duncan et al., 2007). Previously, we described an NLRP3-dependent pathway that is required to induce CD8 T cell IFNγ responses to *T. gondii* infections (Kongsomboonvech et al., 2020). For these reasons, the TG_278878 and TG_278882 NTPases were pursued as candidates in our search of ROCTR.

**Figure 2:**
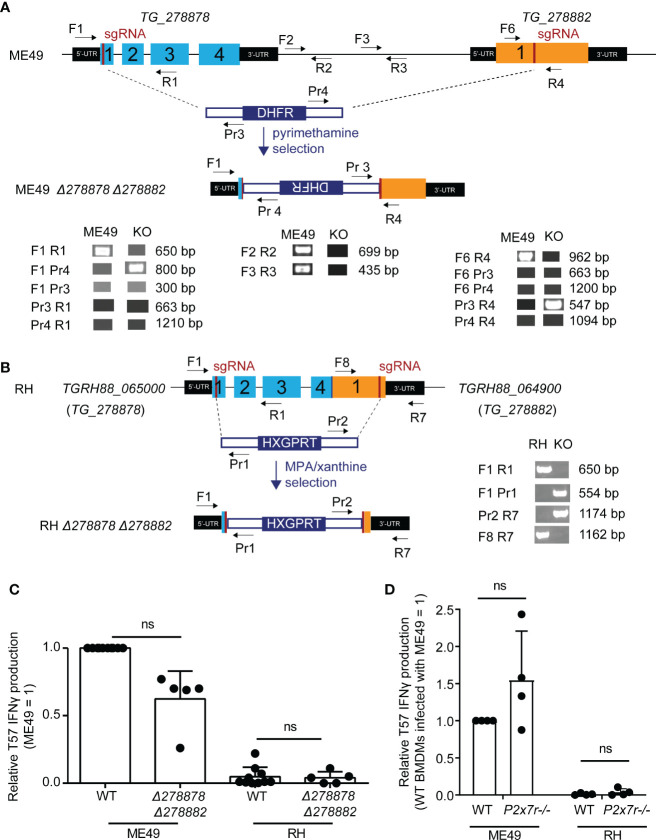
Disruption of two novel NTPases on *Toxoplasma gondii* chromosome XII does not significantly alter the TGD057-specific CD8 T cell IFNγ response to parasite-infected BMDMs. **(A)** QTL chromosome XII candidates, *TG_278878* and *TG_278882*, were deleted in the ME49 *Δhxgprt* (type II) background using CRISPR/Cas9. The strategy included targeting both genes with sgRNAs and repair with a *DHFR* selectable marker as indicated in the schematic. Diagnostic PCR with the specified primers revealed that during non-homologous end joining (NHEJ) repair the *DHFR* cassette integrated in opposite orientation with respect to the two NTPases genes and removed the genetic material internal to the two Cas9 cut sites of the ME49 *Δ278878 Δ278882* strain. **(B)** As in **(A)**, but homology directed repair (HDR) was used to generate a RH *Δ278878 Δ278882* deletion strain in the RH *Δku80 Δhxgprt* genetic background. An *HXGPRT* selection cassette with homology arms integrated in the predicted orientation with respect to the NTPase genes and removed the DNA sequence internal to the two Cas9 cut sites. The RH locus, as shown in supplemental data, is significantly altered in which exon 4 of the RH version of *TG_278878* (*TGRH88_065000*) is fused to an exon 1 truncated *TG_278882* (*TGRH88_064900*) gene. **(C)** ME49 and RH *Δ278878 Δ278882* double deletion strains were assayed for their abilities to induce host CD8 T cell IFNγ responses. The IFNγ level was normalized to that elicited by the wildtype (WT) type II *T. gondii* strain (ME49 = 1), and each dot represents the result from a single experiment. Statistical analysis was performed using a Kruskal-Wallis and *post-hoc* Dunn’s test comparing the deletion to parental strains; ns, non-significant. **(D)** BMDMs with the indicated *P2xr7* deletion (-/-) were infected with ME49 or RH strains. T57 IFNγ responses were normalized to the response elicited by wildtype BMDMs infected with the type II ME49 strain (= 1). Average of 4 experiments +SD is shown, each dot represents the result from an individual experiment. Statistical analysis was performed by one-way ANOVA with Bonferroni’s correction; ns, non-significant.

Double mutant *Δ278878 Δ278882 T. gondii* parasite strains were generated in both the RH *Δhxgprt Δku80* and ME49 *Δhxgprt* genetic backgrounds using CRISPR/Cas9. Parasites were given Cas9 and small guide RNAs (sgRNAs) targeting the first exon of each gene and deletion strains were selected for those that bore evidence of repair with a transfected selectable marker (**Figure 2**). In the type II ME49 deletion strain, both NTPase genes were successfully disrupted and the DNA sequence between the two CRISPR/Cas9 cut-sites was replaced with a dihydrofolate reductase (*DHFR*) selectable marker (*Δ278878::DHFR::Δ278882*) as confirmed by PCR (**Figure 2A**) and sequencing of the edited locus (not shown). During attempts to generate a double deletion strain in the type I RH background, it became clear that the syntenic NTPase genes to *TG_278878* and *TG_278882*, corresponding to *TGRH88_065000* and *TGRH88_064900* respectively, were significantly different from those of type I GT1 and type II ME49 strains. PCR performed with several primer pairs flanking, within and between *TG_278878* and *TG_278882* genes, consistently yielded PCR products indicative of a large-scale deletion between the two genes in the RH genetic background (**Figure S5**). The recent release of the RH genome (GCA_013099955.1) confirms these results and indicates that a 4.5 Kb deletion occurred at this locus, in which the 3’ end of exon 4 for *TG_278878* experienced a 338 bp truncation and was then fused to a *TG_278882* gene missing 1.2 Kb of the 5’ end of exon 1. Whether *TGRH88_065000* and *TGRH88_064900* encode functional NTPases is unknown. Nonetheless, an RH *Δ278878 Δ278882* strain was generated in which an *HXGPRT* selectable marker replaced the DNA sequence internal to the two CRISPR/Cas9 cut sites (**Figure 2B**). However, no difference in the T57 IFNγ response was observed comparing the NTPase deletion strains with their parental counterparts (**Figure 2C**). Finally, *P2x7r*-/- BMDMs were screened to address whether P2X7R contributed to the NLRP3-dependent T57 IFNγ response (Kongsomboonvech et al., 2020), however, the response to parasite-infected *P2x7r*-/- BMDMs is intact (**Figure 2D**), ruling against a major role for exogenous ATP triggered P2X7R signaling in this system. In summary, although there appears to be a slight reduction in the T57 IFNγ response to the ME49 NTPase deletion strain, this difference is not significant. Hence, if these NTPases are ROCTRs, they exert a marginal effect at best, which would be consistent with the small effect QTLs produced by the genetic mapping.

### 3.3 The role of host RIPK3 and the *Toxoplasma gondii* chromosome X ROCTR candidate TgNSM in regulating CD8 T cell IFNγ responses

**Figure 3:**
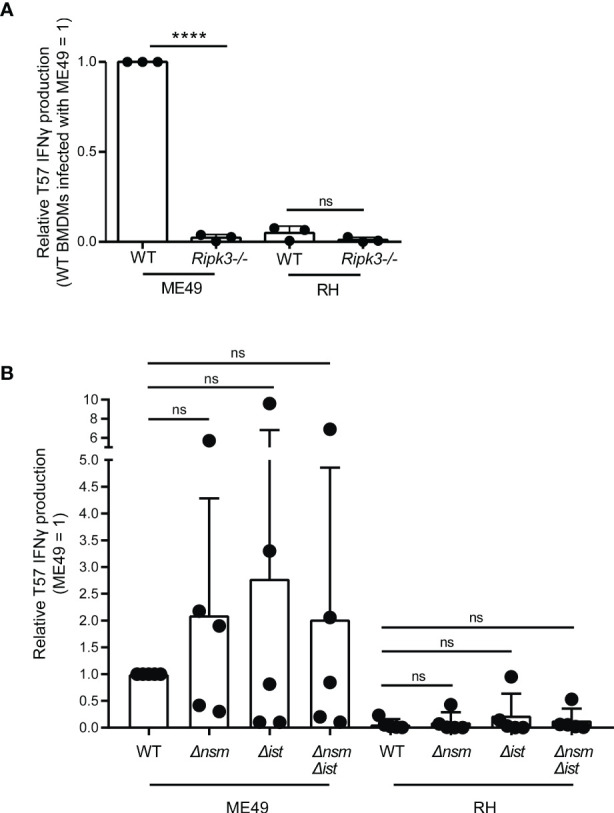
Macrophage expression of RIPK3, but not parasite regulators of necroptosis, is required for eliciting CD8 T cell IFNγ responses to *Toxoplasma gondii*. **(A)** BMDMs with the indicated *Ripk3* deletion (-/-) were infected with ME49 or RH strains. T57 IFNγ responses were normalized to the response elicited by wildtype (WT) BMDMs infected with the type II ME49 strain (= 1). Average of 3 experiments +SD is shown, each dot represents the result from an individual experiment. Statistical analysis was performed by one-way ANOVA with Bonferroni’s correction; ****p < 0.0001; ns, non-significant. **(B)** BMDMs were infected with the indicated WT, *Δnsm, Δist* and double deletion ME49 and RH strains and naïve T57 CD8 T cells IFNγ responses were analyzed. Plotted is the average +SD of 5 experiments. Statistical analysis was performed using a Kruskal-Wallis and *post-hoc* Dunn’s test comparing the parental to deletion strains; ns non-significant.

PEAK2 encompasses multiple genes, however one candidate was intriguingly close to the genetic marker 46.m03675_at7 that produced the maximal LOD score, the dense granule TgNSM. Recently, TgNSM was described to be exported to the host nucleus where it associates with the NCoR/SMRT co-repressor complex promoting the transcriptional repression of many IFN-stimulated genes (Rosenberg and Sibley, 2021). Importantly, TgNSM works together with another exported dense granule, TgIST, to inhibit the transcription of key regulators of necroptosis following IFNγ or IFNβ stimulation (Rosenberg and Sibley, 2021). Necroptosis is a programed cell death response that is mediated by the RIPK3/RIPK1 signaling complex (Pasparakis and Vandenabeele, 2015), and in certain contexts is initiated by IFN-STAT1 signaling (Thapa et al., 2013). Several studies have demonstrated that RIPK3-dependent necroptosis is potent at inducing CD8 T cell activation *in vitro* and *in vivo* (Yatim et al., 2015; Ren et al., 2017; Rana et al., 2020; Aaes and Vandenabeele, 2021). Hence the role of RIPK3 was explored. Importantly, its expression in BMDMs was found to be absolutely required for eliciting T57 IFNγ responses to *T. gondii* (**Figure 3A**). Moreover, TgIST is a potent repressor of STAT1 signaling *via* its recruitment of Mi-2/NuRD to phosphorylated STAT1 dimers (Gay et al., 2016; Olias et al., 2016) and we previously demonstrated STAT1 is a requirement for the T57 IFNγ response (Kongsomboonvech et al., 2020), hinting of a possible STAT1-RIPK3 axis that could be intersected by ROCTRs. Given these observations, single and double TgIST and TgNSM deletion strains were screened. However, no significant difference was observed between parental and deletion strains (**Figure 3B**), indicating TgNSM on chromosome X and TgIST are not ROCTRs. This supposition is supported by previous findings regarding the parasite’s export machinery, which is required for TgIST and TgNSM export from the PV to the host nucleus (Gay et al., 2016; Olias et al., 2016; Rosenberg and Sibley, 2021) but is dispensable for T57 IFNγ responses to *T. gondii* (Kongsomboonvech et al., 2020). Thus, while STAT1- and RIPK3-dependent processes are necessary for inducing CD8 T cell IFNγ responses to *T. gondii*, any modulation of these signaling pathways by specific *T. gondii* effectors can be overcome by host macrophages to support T cell activation and/or differentiation in this system.

### 3.4 A minor QTL peak on *T. gondii* chromosome X identifies a group of dense granules that regulate host CD8 T cell IFNγ responses

**Figure 4:**
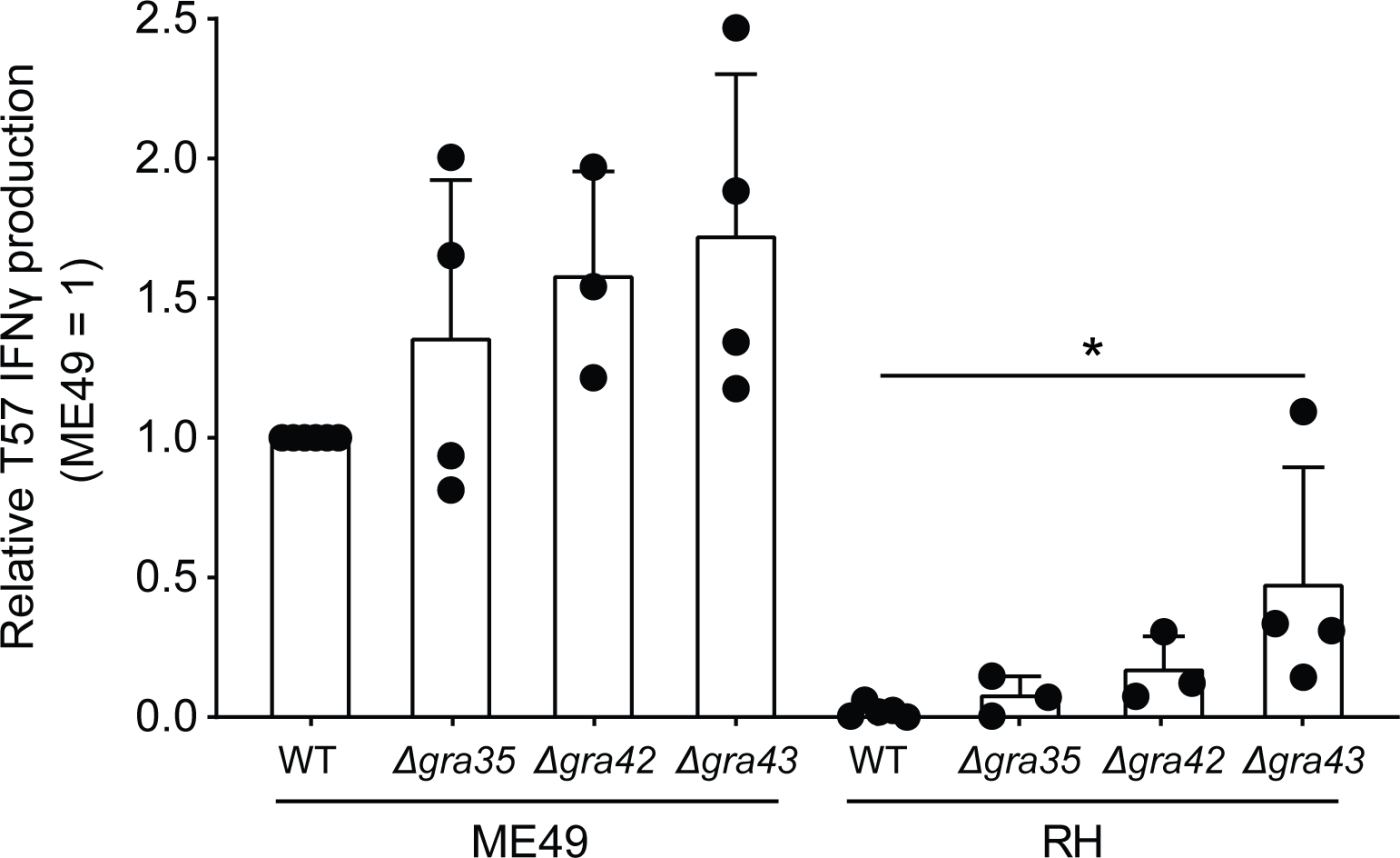
*Toxoplasma gondii* GRA43 regulates TGD057-specific CD8 T cell IFNγ responses to parasite-infected BMDMs. BMDMs were infected with WT, *Δgra35*, *Δgra42*, or *Δgra43 T. gondii* strains as indicated and the naïve T57 CD8 T cell IFNγ response was measured. Average +SD of 3-4 experiments is plotted; each dot represents the result from an individual experiment. Statistical analysis was performed using a Kruskal-Wallis and *post-hoc* Dunn’s test comparing the parental to deletion strains, only significant values are shown; *p ≤ 0.05.

The genetic marker 42.m03493_at7 that corresponds to PEAK1 is within the gene encoding the *T. gondii* dense granule GRA35. GRA35 was previously identified as an NLRP1 inflammasome activator in *T. gondii* infections of Lewis rat macrophages (Wang et al., 2019). GRA35 localizes to the PVM with the aid of GRA42 and GRA43 and remains in PV lumen in their absence (Wang et al., 2019). Therefore, all three dense granules were analyzed and the T57 IFNγ response to *Δgra35*, *Δgra42*, and *Δgra43* deletion strains was measured as before. Compared to the parental RH strain, CD8 T cell IFNγ responses were slightly elevated to BMDMs infected with RH *Δgra35* and RH *Δgra42* strains, but significant differences were only observed in response to RH *Δgra43* infections (**Figure 4**). A similar trend was observed in the type II ME49 genetic background, but none of the differences between deletion and wildtype strains were of statistical significance (**Figure 4**). Given the relatively small effect that GRA35 had on the phenotype, we did not pursue the generation of complementation strains to address whether GRA35 is a ROCTR accounting for some of the strain-differences in the T57 IFNγ response. Regardless, the upstream regulator of GRA35, GRA43, seems important for modulating the T57 IFNγ response to *T. gondii*.

### 3.5 ASP5 and GRA43 do not impact PV localization nor processing of TGD057

**Figure 5:**
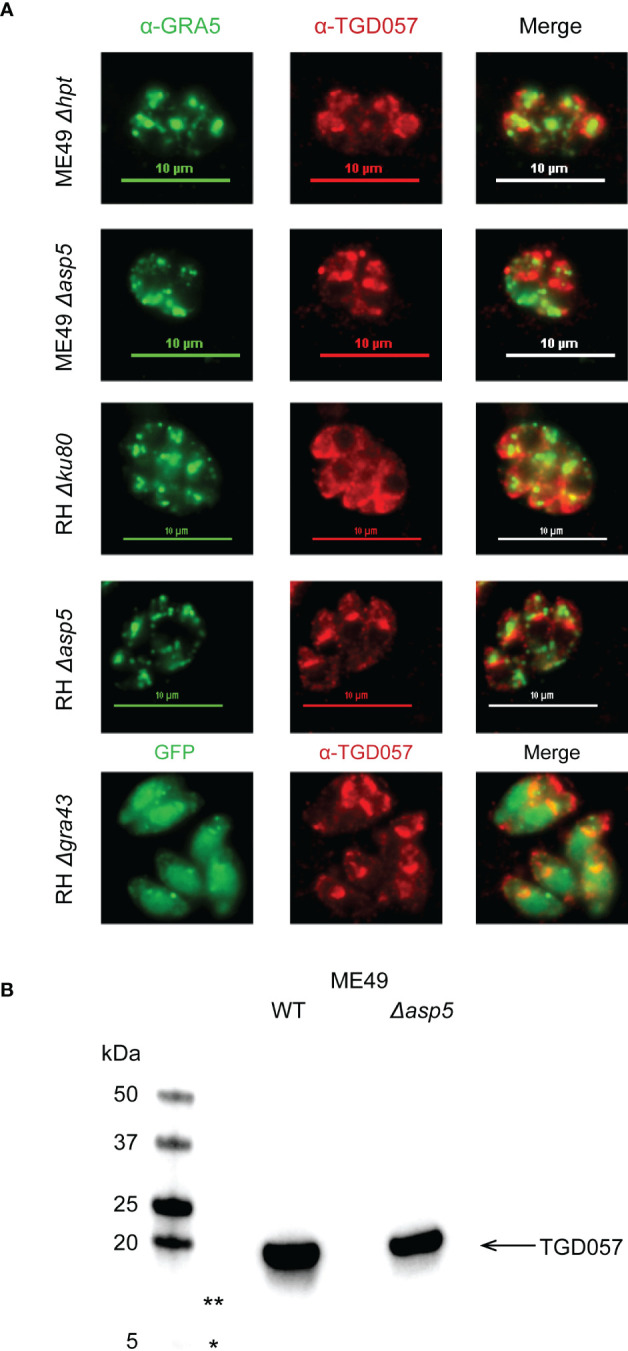
ASP5 and GRA43 do not impact PV localization nor processing of TGD057. **(A)** Human foreskin fibroblasts were infected with the indicated *T. gondii* strains. After 16 hours of infection, the samples were fixed and visualized by immunofluorescence. TGD057 was detected with a rabbit polyclonal α-TGD057 antibody, visualized in red for all strains. The PV is indicated by the PVM integral and PV luminal dense granule GRA5, visualized in green for all strains except RH *Δgra43* which is GFP+. A representative immunofluorescence image from 2 experiments is shown. **(B)** TGD057 from lysates of *T. gondii* parental and *Δasp5* strains were detected by western blot analysis using an α-TGD057 polyclonal antibody. TGD057 is 18 kDa. Representative blot of two experiments is shown. Asterisks indicate 13 kDa (**) and 5 kDa (*) regions on the gel that would correspond to the predicted peptide fragment sizes generated if ASP5 cleaved TGD057 at its putative TEXEL sequence but was not observed.

Collectively, our data suggests that when removing members of the parasite’s PVM-targeting pathway, including the Golgi-resident protein aspartyl protease, ASP5 (Coffey et al., 2015; Hammoudi et al., 2015) and GRA43 (Wang et al., 2019), the host CD8 T cell IFNγ response increases (Kongsomboonvech et al., 2020) (**Figure 4**). It has also been demonstrated for the PV-associated antigen, GRA6, that PVM targeting increases access into the host’s MHC I antigen processing pathway (Lopez et al., 2015; Jensen, 2016). Hence, we tested whether ASP5 or GRA43 affect where TGD057 localizes within the PV. Thus far, the Blanchard and Yap groups and our studies have shown through immunofluorescence assays (IFA) and/or differential centrifugation techniques that TGD057 is a PV resident protein within the PV lumen (Lopez et al., 2015; Kongsomboonvech et al., 2020) and within insoluble structural elements of the tachyzoite (Wilson et al., 2010). Here, the localization of TGD057 in *Δasp5* and *Δgra43 T. gondii* strains was investigated. TGD057 was detected with a rabbit polyclonal α-TGD057 and the PVM and dense granules were marked by α-GRA5. RH *Δgra43* expresses GFP hence GRA5 was not assessed in this strain. Regardless, TGD057 appeared as puncta and was distributed throughout the PV and tachyzoite in all strain types analyzed, irrespective of whether they are stimulatory (ME49, ME49 *Δasp5*, RH *Δgra43*) or not (RH, RH *Δasp5*) (**Figure 5A**). These results imply GRA43 and ASP5 regulate T57 IFNγ responses independently of where TGD057 localizes within the vacuole.

Protein export from the PV is dependent on the parasite’s Golgi protease ASP5 (Coffey et al., 2015; Hammoudi et al., 2015; Curt-Varesano et al., 2016) and the MYR1 PVM-associated translocation machinery (Franco et al., 2016). As noted above, while this machinery was dispensable for T57 IFNγ response induction, ME49 *Δasp5* elicited a much greater response compared to its parental control (Kongsomboonvech et al., 2020). We previously noted that TGD057 possesses a TEXEL motif and argued from the literature that TGD057 bore no evidence for ASP5-mediated cleavage to assist antigen processing (Kongsomboonvech et al., 2020). To test this, we performed a simple western blot comparing wildtype and *Δasp5* strains using the polyclonal α-TGD057 which recognizes both an N- and C-terminal peptide sequence flanking the putative ASP5 cleavage site of TGD057 (personal communication, Nicolas Blanchard, INSERM). Consistent with initial characterizations of this protein (Wan et al., 2004), TGD057 migrates at the expected size of 18 kDa. Furthermore, no peptide fragments of 5 and 13 kDa were observed, which would be generated if TGD057 were to be cleaved at its putative TEXEL sequence, nor did the signal intensity of TGD057 change in *Δasp5* relative to parental strains (**Figure 5B**, not shown). In summary, the role for ASP5 and GRA43 in this system is not to process nor localize TGD057 within the PV. Instead, their ability to target dense granules to the PVM may assist the localization of unidentified ROCTRs to the PVM, or regulate the PVM integrity, thereby controlling CD8 T cell activation phenotypes.

### 3.6 Polymorphic ROP16 regulates the early IFNγ transcriptional response of CD8 T cells

**Figure 6:**
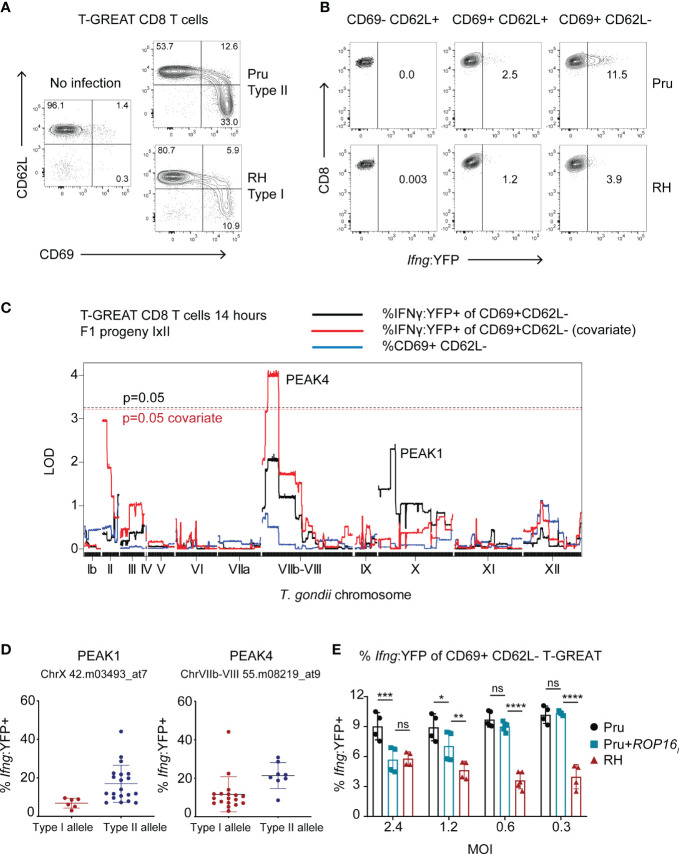
Genetic mapping identifies ROP16 as a modulator of the early *Ifng* transcriptional response of activated CD8 T cells. **(A)** Naïve T-GREAT CD8 T cells were co-cultured with BMDMs or BMDMs infected with the indicated type II Pru or type I RH *T. gondii* strains. At 14 hours, CD3+ CD8+ T-GREAT cells were analyzed for expression of CD62L and CD69 by flow cytometry. Representative dot plots are shown (N = 10 experiments); numbers are the frequency of CD3+ CD8+ T GREAT cells that fall within the indicated gates. **(B)** Representative dot plots (N = 9 experiments) indicate the frequency of CD3+ CD8+ T-GREAT cells that express the *Ifng :* YFP reporter. Following activation, naïve T cells (CD62L+ CD69-) first upregulate CD69 (CD69+ CD62L+) and then shed CD62L from their surface (CD69+ CD62L-); numbers are the frequency of YFP+ cells that fall within the depicted gates at the various stages of activation. **(C)** QTL analysis was performed for the following phenotypes: frequency of activated (% CD69+ CD62L-) T-GREAT CD8+ T cells (blue line), and frequency of *Ifng* transcript positive cells among the activated subset of T-GREAT CD8+ T cells (% *Ifng :* YFP+ of CD69+ CD62L-) (black line) following 14 hours of co-culture with F1 IxII progeny. Covariate QTL analysis for the % *Ifng :* YFP+ phenotype is also shown (red line), which returned a significant QTL surpassing genome wide permutation testing (p = 0.05, dashed red line). Threshold values for the primary scan of the % *Ifng :* YFP+ phenotype are also indicated (p = 0.05 black dashed line), which did not return a significant QTL. **(D)** Allelic effect plots for the genetic markers corresponding to PEAKS 1 and 4, are shown. The frequency of *Ifng :* YFP+ cells among total CD69+ CD62L- CD8+ T-GREAT cells is shown. Each dot represents the value obtained for individual F1 IxII parasite strains. **(E)** Frequency of *Ifng :* YFP+ cells among total CD69+ CD62L- CD8+ T-GREAT cells following 14-18 hours of co-culture with BMDMs infected with the indicated parasite strains. Each symbol represents the result from an independent experiment and multiple MOIs were assessed for RH, Pru and Pru+*ROP16_I_ T. gondii* strains, the latter of which expresses the type I *ROP16* allele as a transgene. Statistical analysis was performed with two-way ANOVA with Bonferroni’s correction; ****p <0.0001, ***p < 0.001, **p < 0.01, *p < 0.05, ns, non-significant. Pru is significantly different from RH at each MOI; p < 0.001, not indicated on plot.

Following initial T cell receptor (TCR) stimulation by antigens (also known as ‘signal 1’), early activated T cells require additional signals including co-stimulation (‘signal 2’) and soluble factors (‘signal 3’) to initiate the production of cytokines like IFNγ. Since ROCTRs may potentially intersect each of these steps and to assist genetic mapping, CD8 T cell IFNγ differentiation was measured at an earlier stage and disentangled from phenotypes associated with T cell activation using T-GREAT CD8 T cells. T-GREAT mice encode the same T57 TCR and report IFNγ transcription by YFP fluorescence as measured by flow cytometry, which can be detected as early as 14-18 hours after activation by parasite-infected BMDMs (Kongsomboonvech et al., 2020). Importantly, *Ifng* transcription can be measured independently of CD69 expression, which is a proxy for early TCR signaling events mediated by MHC antigen presentation (Cebrián et al., 1988) and TGD057 release from the vacuole. Therefore, T-GREAT cells were used to distinguish between *T. gondii* ROCTRs that may regulate activation (i.e. CD69+) from those that regulate differentiation (*Ifng :* YFP+) of CD8 T cells. Naïve T-GREAT cells predominately express CD62L, which promotes lymphocyte homing to secondary lymphoid organs (Steeber et al., 1996), and do not express CD69, which retains activated cells within the secondary lymph organs by antagonizing S1PR1 (Shiow et al., 2006) (**Figure 6A**). In contrast, at 14 hours of co-culture with infected BMDMs, T-GREAT cells significantly upregulate CD69 and downregulate CD62L, which occur more readily in response to type II compared to type I strains (**Figures 6A**, **S6A**). Type II strains are also better at inducing the early *Ifng* transcriptional response which is mainly observed in the fully activated CD69+ CD62L- subset compared to the CD69+ CD62L+ or CD69- CD62L+ populations of T-GREAT cells (**Figures 6B**, **S6B**, not shown). Hence, the frequency of CD69+ CD62L- T-GREAT cells (**Figure S6A**) and *Ifng :* YFP+ among activated CD69+ CD62L- T-GREAT cells (**Figure S6B**) were measured in response to F1 progeny of the IxII cross and genetic mapping was performed. Although QTLs with an LOD > 2 were not found for the CD69+ T-GREAT activation phenotype, two peaks were identified for the *Ifng :* YFP+ phenotype, including a small QTL with the same boundaries as PEAK1 identified for the T57 IFNγ response at 48 hours (TGME49_chrX:1383691-1802907), and a second QTL observed on chromosome VIIb-VIII (“PEAK4”, TGME49_chrVIIb-VIII: 796459-2341638) (**Figure 6C**). Importantly, covariate analysis revealed that PEAK4 is significant with an LOD = 4. 1 (additive-QTL, p < 0.05). No evidence is inferred for epistatic interactions between PEAK4 and PEAK1 (**Figure S2B**). Effect plots reveal that type I, compared to type II alleles, for both QTLs correlate with lower *Ifng* transcriptional responses of T-GREAT cells (**Figure 6D**).

PEAK4 encompasses a large 1.54 Mb region of 208 genes, with several putative dense granules and rhoptry proteins as ROCTR candidates, including polymorphic ROP16 (**Table 1**). Type I and III alleles of ROP16 are known to induce the alternative activation (M2) program of infected BMDMs (Jensen et al., 2011) and can inhibit CD8 T cell expansion and IFNγ production *in vivo* (Tuladhar et al., 2019; Chen et al., 2020) and *in vitro* (Kongsomboonvech et al., 2020). Importantly, a single polymorphism in the ligand binding domain renders the type II allele unable to maintain STAT6 and STAT3 (Yamamoto et al., 2009). Therefore, we hypothesized that ROP16 is the ROCTR associated with the PEAK4 QTL. A type II Pru strain expressing the *ROP16_I_* allele from the type I strain (Pru+*ROP16_I_*) was analyzed, as it induces M2 BMDMs and activates the aforementioned STATs to regulate a variety of immune-related genes (Saeij et al., 2007; Jensen et al., 2011; Jensen et al., 2013; Chen et al., 2020). In this system, ROP16 mediates an MOI-dependent effect on the early T-GREAT *Ifng* transcriptional response to *T. gondii* (**Figure 6E**). At higher MOIs the *Ifng* transcriptional response is decreased by Pru+*ROP16_I_*, particularly at MOI 2.4, to levels that resemble those induced by the type I RH strain. In contrast, no discrepancy is observed between Pru+*ROP16_I_* and its parental strain at lower MOIs of 0.6 and 0.2. Whether the MOI-dependent effect of ROP16 diminishes the phenotype-genotype correlation at PEAK4 is unclear. However, these results are consistent with our previous observations that Pru+*ROP16_I_* reduces IFNγ secretion by T57 cells at 48 hours (Kongsomboonvech et al., 2020).

## 4 Discussion

*Toxoplasma gondii* is considered a successful parasite because it can infect nearly all warm-blooded vertebrates. To accommodate this broad host range, the parasite co-evolves with its host to be able to modulate host immune responses. Among various host immune cells, CD8 T cells are critical for the elimination of parasites. Therefore, we hypothesized the existence of *T. gondii* virulence factors, or ROCTRs, responsible for strain-specific differences in inducing CD8 T cell responses to infection. Broadly, the identity of ROCTR would help us better understand how *T. gondii* can survive in a variety of hosts that it infects.

The search for ROCTR was underpinned by our earlier analysis of CD8 T cell IFNγ responses to multiple atypical (haplogroups IV-X), Eurasian (types I, II, III) and North American strains (haplogroups XI-XII), which revealed only clade A strains (type I, haplogroups VI and VII) were low inducers of T57 CD8 IFNγ production (Kongsomboonvech et al., 2020). To identify ROCTR, we performed QTL analysis using progeny from three separate genetic crosses between the clonal lineage strains and made gene deletions or screened transgenic parasites for selected ROCTR candidate genes, representing an analysis of 120 strains in this system (**Table S1**). Analyzing the T57 IFNγ response at 48 hours, three QTLs of small effect on *T. gondii* chromosomes X and XII were revealed by F1 progeny from the IxII cross, that were not corroborated by an analysis of F1 progeny from the IIxIII and IxIII genetic crosses. Consequently, we propose there are multiple polymorphic ROCTRs, each with small effect, that possibly account for the clade A-specific strain-differences in CD8 T cell IFNγ production. We also believe our search for ROCTR was inhibited by the complexity of the phenotype measured—the CD8 T cell IFNγ response—which requires intricate host processes related to MHC I antigen presentation, co-stimulation by co-receptors and ligands, and differentiation steps, each of which may be intersected by parasite effectors. Indeed, multiple dense granules and rhoptry proteins have been shown to modulate CD8 T cell activation, including but not limited to GRAs -2, -3, -4, -6, -7, -15, -24 and ROPs -5, -16, -18 (Yamamoto et al., 2011; Lopez et al., 2015; Rommereim et al., 2019; Chen et al., 2020; Kongsomboonvech et al., 2020).

Therefore, to refine genetic mapping we decided to reassess CD8 T cell differentiation at an earlier time point following activation using the *Ifng :* YFP reporter T-GREAT system, which revealed ROP16 as a ROCTR. These results are consistent with reports from other laboratories which similarly found that ROP16 thwarts full expansion of TGD057-specific cells *in vivo* (Tuladhar et al., 2019; Chen et al., 2020). Type I and III alleles of ROP16 regulate over 900 genes in infected BMDMs, many of which can potentially repress T cell responses including ROP16-dependent induction of co-inhibitory receptors (PD-L1, PD-L2), and the suppression of cytokines (IL-12, IL-23) and co-stimulatory receptors (CD70) (Jensen et al., 2011; Jensen et al., 2013; Chen et al., 2020). The MOI-dependent effect may indicate that multiple ROP16-induced immune genes only work at higher concentrations or expression levels in the infected macrophage. Moreover, given the MOI-dependent effect of ROP16, other parasite genes that enhance parasite survival in this co-culture system may assist *T. gondii*-specific regulation. Recently, the PEAK1 candidate, GRA35, was reported to promote parasite survival in IFNγ-stimulated human HFFs in a type II allele dependent fashion (Lockyer et al., 2022). Similarly, ROP39, which is also encoded within the PEAK4 QTL and is highly polymorphic (**Table 1**), was recently described as a virulence factor that antagonizes mouse Irgb10 (Singh et al., 2023). We have not explored the allelic impact of ROP39 and GRA35 in our system, but their polymorphisms may intersect the early *Ifng* transcriptional response of CD8 T cells, either through promoting parasite survival in this context or regulating an unknown pathway that mediates this phenotype.

Although we were unable to leverage our genetic mapping to identify other ROCTRs, several observations were made along the way that hint at their potential function and the overall requirements for naïve CD8 T cell differentiation to become IFNγ producers. First, clues as to where ROCTRs might function come from studies of parasites that are defective in the PVM-targeting pathway of dense granules. The PVM targeting factor, GRA43, had a significant effect at repressing T57 IFNγ production (**Figure 4**). Similarly, ASP5 represses CD8 T cell IFNγ responses when expressed in the ME49 but not RH backgrounds (Kongsomboonvech et al., 2020). As both ASP5 and GRA43 have PVM-targeting functions for various GRAs (Coffey et al., 2015; Hammoudi et al., 2015; Wang et al., 2019), we hypothesize they may regulate the CD8 T cell response by either, 1) shuttling ROCTRs to the PVM where they interact with the host cell compartments, and/or, by 2) modulating the localization of PVM-targeted proteins that maintain PVM integrity.

Second, our interrogation of the chromosome X PEAK2, led us to test the role of RIPK3 in CD8 T cell responses. We were intrigued by the possibility that TgNSM, whose function includes the inhibition of necroptosis and the repression of a select number of IFN-stimulated genes (Rosenberg and Sibley, 2021), was the ROCTR underpinning PEAK2. However, the function of this dense granule requires protein export *via* the MYR1 translocon machinery (Rosenberg and Sibley, 2021) and T57 IFNγ responses are *independent* of MYR1 (Kongsomboonvech et al., 2020). Hence, it is perhaps not surprising that conclusive evidence was lacking for TgNSM or the related TgIST in regulating this phenotype. In contrast, macrophage expression of RIPK3 was found to be absolutely required for CD8 T cell IFNγ production to *T. gondii* (**Figure 3**). It is well established that necrotic cell death is a potent inducer of T cell responses (Gallucci et al., 1999; Shi et al., 2000) and RIPK3-dependent necroptosis is especially effective for eliciting anti-tumor immunity [reviewed in (Aaes and Vandenabeele, 2021)]. Such immunogenicity has been linked to the release of damage associated molecular patterns (DAMPs), primarily those of ATP (Elliott et al., 2009; Michaud et al., 2011; Martins et al., 2014) and HMGB1 (Apetoh et al., 2007; Yamazaki et al., 2014), but also the surface expression of calreticulin (Obeid et al., 2007; Martins et al., 2011). In some models of RIPK3-mediated immunogenicity, it is the ‘necrosome’-induced NFKB-dependent cytokine release that promotes optimal CD8 T cell cytolytic responses (Yatim et al., 2015). In mouse models of *T. gondii* infection, RIPK3 promotes certain aspects of small intestinal pathology that occurs following oral infection and clearance of tissue cysts during chronic infection (Cervantes Patrick et al., 2021), processes that are driven by T cell immunity (Liesenfeld et al., 1996; Suzuki, 2020). Therefore, RIPK3 is likely involved in shaping adaptive immune responses to *T. gondii*, as directly measured here.

Third, we analyzed a set of previously uncharacterized of NTPases on chromosome XII, whose locus underwent a significant truncation in the RH strain (**Figure S5**). We speculated that parasite NTPases might modulate T cell function through scavenging and hydrolysis of ATP, including inhibiting of inflammasome activation. Extracellular ATP (eATP) induces signaling through P2X7 receptors (P2X7R), leading to potassium (K+) efflux and NLRP3 inflammasome activation (Jo et al., 2016; Amores-Iniesta et al., 2017). Exogenous ADP has also been shown to mediate NLRP3 inflammasome activation through P2Y2 purinergic receptors (Baron et al., 2015), host receptors which were not explored here. In addition, depletion of cytosolic ATP by *T. gondii*, as previously shown for the related NTPase I (Olias et al., 2016), could potentially thwart NLR-oligomerization and inflammasome activation which is an ATP-dependent process (Duncan et al., 2007). Putative modulation of NLRP3 activity by the chromosome XII NTPases would coincide with our previous finding that an NLRP-dependent pathway is required for the commitment of activated CD8 T cells to differentiate into IFNγ-producing cells (Kongsomboonvech et al., 2020). However, we found no evidence to support this hypothesis. Whether the expansion of parasite GDA1/CD39 family of ecto-ATPases has masked the specific effect 278878 and 278882 NTPases in our system is unknown. Expression of CD39 ecto-ATPases are determinants of cancer immune evasion (Michaud et al., 2012), and T cells themselves are modulated by eATP (Borges da Silva et al., 2018), hence it remains an outstanding question as to what role, if any, does the collective activity of all the *T. gondii* CD39 ecto-ATPases play in immune modulation.

Finally, our results can rule out certain mechanisms that may account for strain-differences in manipulating the naïve CD8 T cell IFNγ response. First, any distinguishing feature between RH and GT1 type I strains, which are divergent in terms of the rate of parasite replication and extracellular survival, and immune evasion during a secondary infection (Jensen et al., 2015), are likely to be uninvolved. Second, our genetic mapping fails to identify even weak QTLs at the *ROP5* and *TGD057* loci. This was expected for *TGD057* as this gene is conserved between clonal lineage strains, but *ROP5* is a highly duplicated and polymorphic locus (Xia et al., 2021) that accounts for parasite strain-differences in mouse virulence (Behnke et al., 2011; Reese et al., 2011). Previously, we demonstrated that *Δrop5* strains complemented with avirulent type I and II *ROP5A* alleles, which are unable to antagonize known effector IRGs (Howard et al., 2011; Reese et al., 2014), were able to equally inhibit the CD8 T cell IFNγ response compared to the wildtype type I strain (Kongsomboonvech et al., 2020). This finding is consistent with our QTL mapping data and suggests that all *ROP5* alleles are functional repressors of CD8 T cell activation, but likely require assistance from another genetic determinant within the type I genetic background to perform its function. Perhaps ROP5A, which currently has no known function or interacting partner, requires an unidentified ROCTR to effectively inhibit MHC I antigen presentation, thereby thwarting CD8 T cell IFNγ responses.

In summary, the CD8 T cell IFNγ response to *T. gondii* infections is a complex phenotype that is the derivative of multiple processes, including antigen presentation, CD8 T cell activation, and differentiation. To avoid being eliminated by host cytotoxic CD8 T cells and ensure its survival, it is possible *T. gondii* manipulates all or one of the steps required for this response, likely through ROCTRs, as well as maintain its intact PV. We hypothesize that multiple ROCTRs may intersect these pathways, providing insights to novel host-parasite interactions that control CD8 T cell immunity.

## Data availability statement

The original contributions presented in the study are included in the article/**Supplementary Material**. Further inquiries can be directed to the corresponding author.

## Ethics statement

All animal protocols were reviewed and approved by UC Merced’s Committee on Institutional Animal Care and Use Committee (AUP 20-0015). All mouse work was performed in accordance to the Guide to the Care and Use of Laboratory Animals of the National Institutes of Health and the Animal Welfare Act (assurance number A4561-1).

## Author contributions

Conceptualization: AK, LL, KJ. Formal analysis: AK, LL, KJ. Funding acquisition: KJ. Investigation: AK, LL, FN, FR, SS, KJ. Methodology: AK, LL, FN, FR, SS, KJ. Project administration: AK, LL, KJ. Resources: AR, KJ. Supervision: KJ. Validation: AK, LL, FN, FR, KJ. Visualization: AK, LL, FN, FR, KJ. Writing – original draft: AK, KJ. Writing – review & editing: AK, LL, FN, KJ. All authors contributed to the article and approved the submitted version.

## Supplementary material

Supplementary Figure 1 TGD057-specific CD8 T cell IFNγ responses to individual *Toxoplasma gondii* F1 progeny of the type I x type II cross. TGD057-specific CD8 T cell responses to *T. gondii*-infected BMDMs were assayed, as previously described in **Figure 1**, with F1 progeny of the type I x II cross (F1 IxII). The IFNγ concentration at 48 hours was measured by ELISA and normalized to that of the type II strain. Average of 2-5 experiments +SD is plotted for each strain and values are indicated above the bar graphs; each dot represents the result from an individual experiment.

Supplementary Figure 2 A two-dimensional (2D) QTL genome-wide scan does not detect interactive effects between loci associated with CD8 T cell IFNγ responses to *T. gondii* infections. **(A)** 2D QTL analysis of T57 IFNγ secretion at 48 hours to *T. gondii* F1 IxII infections. The selected *T. gondii* chromosomes are labeled on both axes. The LOD scores in the 2D plot are represented on a color scale indicated on the right. The LOD score scale from 0 to 4.2 corresponds to the upper left triangle which compares the full two-locus or “interactive QTL” model, in which all possible combinations of two markers are calculated for epistasis, to an additive-QTL model. The LOD score scale from 0 to 4.8 corresponds to the lower right triangle of the 2D plot, which compares the two-locus to a single-locus QTL model. No two pairs of loci in the two-locus model surpass a p-value of p = 0.1 (LOD > 8), and no advantage is gained over a single-QTL or additive-QTL model (R/qtl). **(B)** As in **(A)**, but 2D QTL analysis of the *Ifng :* YFP+ frequency of CD69+ CD62L- T-GREAT T cells at 14 hours of co-culture with F1 IxII infectioned BMDMs is plotted. No two pairs of loci in the two-locus model surpass a p-value of p = 0.1, and no advantage is gained over a single-QTL or additive-QTL model (R/qtl).

Supplementary Figure 3 Genetic linkage analysis of TGD057-specific CD8 T cell IFNγ responses to *Toxoplasma gondii* F1 progeny of the type I x III cross reveals no significant QTLs. **(A)** T57 CD8 T cell IFNγ responses to BMDM infections with F1 IxIII *T. gondii* strains were measured as previously described in **Figure 1** and normalized to that induced by the type III CEP strain. The normalized values are indicated above the bar graphs. Average of 2 experiments +SD are shown, each dot represents the results from one experiment. **(B)** A genome-wide QTL scan of the CD8 T cell IFNγ response to F1 IxIII *T. gondii*-infected BMDMs was performed. The running LOD score for each genetic marker is shown; *T. gondii* chromosomes are indicated. Significant threshold LOD values of p = 0.05 and p = 0.10 following 1,000 permutations are indicated in red and black, respectively.

Supplementary Figure 4 Genetic linkage analysis of TGD057-specific CD8 T cell IFNγ responses to *Toxoplasma gondii* F1 progeny of the type II x III cross reveals no significant QTLs. **(A)** F1 IIxIII *T. gondii* strains were assayed for T57 CD8 T cell IFNγ responses as described in **Figure 1**. Plotted is the average IFNγ concentration in the supernatant at 48h post addition of T57 CD8 T cells +SD of 3 technical replicates from a single experiment. **(B)** A genome-wide QTL scan of the CD8 T cell IFNγ response to F1 IIxIII *T. gondii* BMDM infections was performed and the running LOD score for each *T. gondii* genetic marker is shown. The significant threshold LOD values of p = 0.05 and p = 0.10 following 1,000 permutations are indicated in red and black, respectively.

Supplementary Figure 5 A large-scale deletion occurred in the NTPase locus of the RH genetic background. **(A)** Schematic of the NTPase locus encoding the genes *TG_278878* and *TG_27888*2 in the ME49 (type II), GT1 and RH (type I) genetic backgrounds. *TGRH88_065000* and *TGRH88_064900* are syntenic to *TG_278878* and *TG_278882*, respectively. The RH genome (GCA_013099955.1) reveals a 4.5 Kb deletion occurred at this locus, in which the 3’ end of exon 4 for *TG_278878* experienced a 338 bp truncation and was then fused to a *TG_278882* gene missing 1.2 Kb of the 5’ end of exon 1. The fusion event is indicated by dashed lines. **(B)** PCR results obtained with the indicated primers in **(A)**, confirms this deletion within RH *Δku80 Δhxgpr*t, but not GT1 and ME49 *Δhxgpr*t parasite strains.

Supplementary Figure 6 CD69 and *Ifng* expression profiles of T-GREAT CD8 T cells in response to individual *Toxoplasma gondii* F1 progeny of the type I x type II cross. **(A)** TGD057-specific T-GREAT CD8+ T cell responses to *T. gondii*-infected BMDMs were assayed at 14 hours, as described in **Figure 6**, with F1 progeny of the type I x II cross (F1 IxII). Frequency of activated CD69+ CD62L- cells among total CD3+ CD8+ T-GREAT cells is plotted. **(B)** As in **(A)**, but the frequency of *Ifng :* YFP+ cells among total CD69+ CD62L- CD3+ CD8+ T-GREAT are shown. Results are from an individual experiment; values are indicated above the bar graphs.

Supplementary Table 1 Parasite strains. The *T. gondii* strains used in this study are grouped in various categories: F1 IxII (F1 progeny of type I GT1 x type II sexual cross); F1 IIxIII (F1 progeny of type II x type III sexual cross); F1 IxIII (F1 progeny of type I x type III sexual cross); parental and gene edited parasites. Genotypes, alternative names, source, and primary references are indicated.

Supplementary Table 2 Oligos and plasmids. Plasmids and the sequences of oligos used in this study, and their purposes are commented upon. Primary references are indicated.
