# Supplementary figures and images for: Variation in CD8 T cell IFNγ differentiation to strains of Toxoplasma gondii is characterized by small effect QTLs with contribution from ROP16
Source: Front Cell Infect Microbiol. 2023 May 23;13:1130965. doi: 10.3389/fcimb.2023.1130965 (PMC10242045; doi:10.3389/fcimb.2023.1130965)

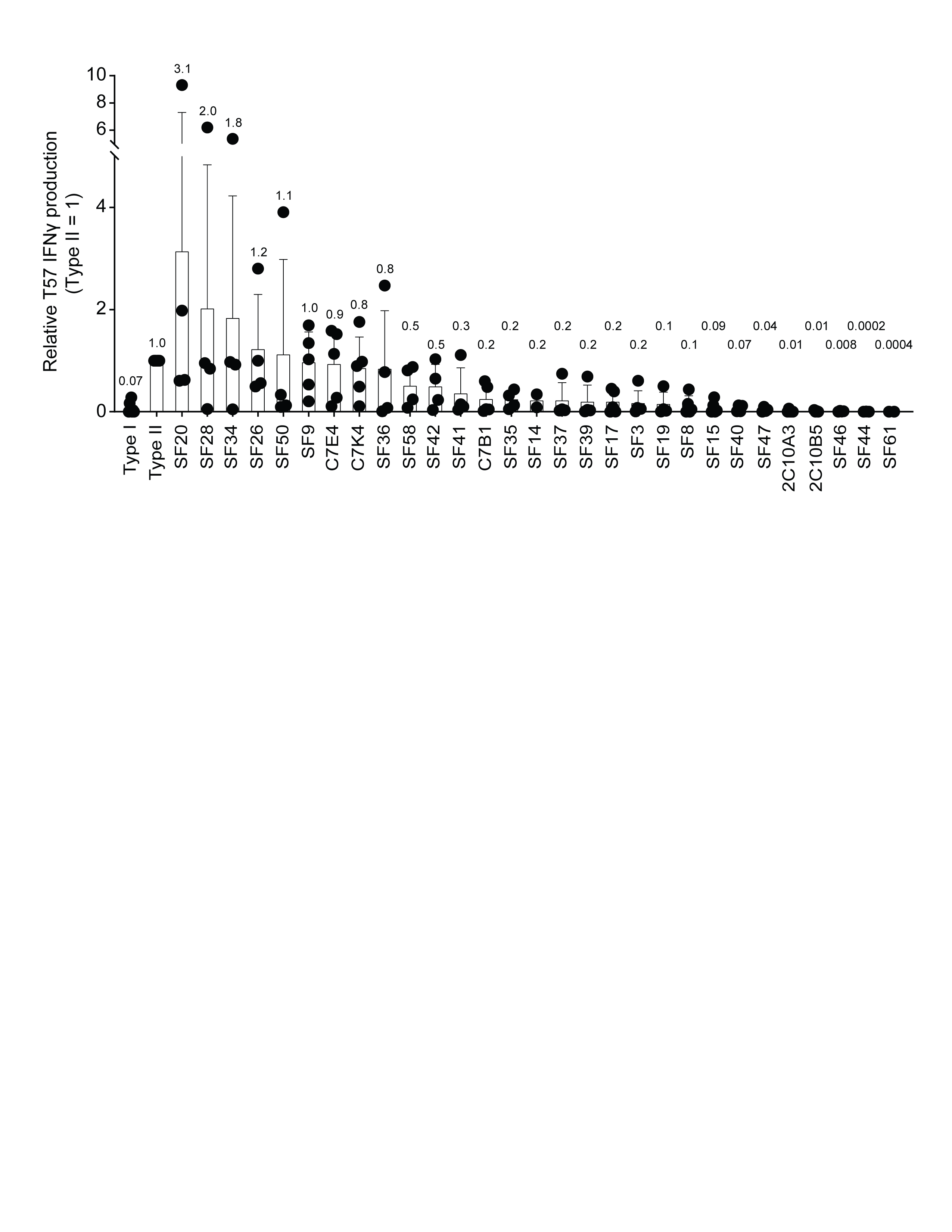

Supplement: Supplementary Figure 1 — TGD057-specific CD8 T cell IFNγ responses to individual Toxoplasma gondii F1 progeny of the type I x type II cross. TGD057-specific CD8 T cell responses to T. gondii-infected BMDMs were assayed, as previously described in Figure 1 , with F1 progeny of the type I x II cross (F1 IxII). The IFNγ concentration at 48 hours was measured by ELISA and normalized to that of the type II strain. Average of 2-5 experiments +SD is plotted for each strain and values are indicated above the bar graphs; each dot represents the result from an individual experiment. [file Image_1.tif]

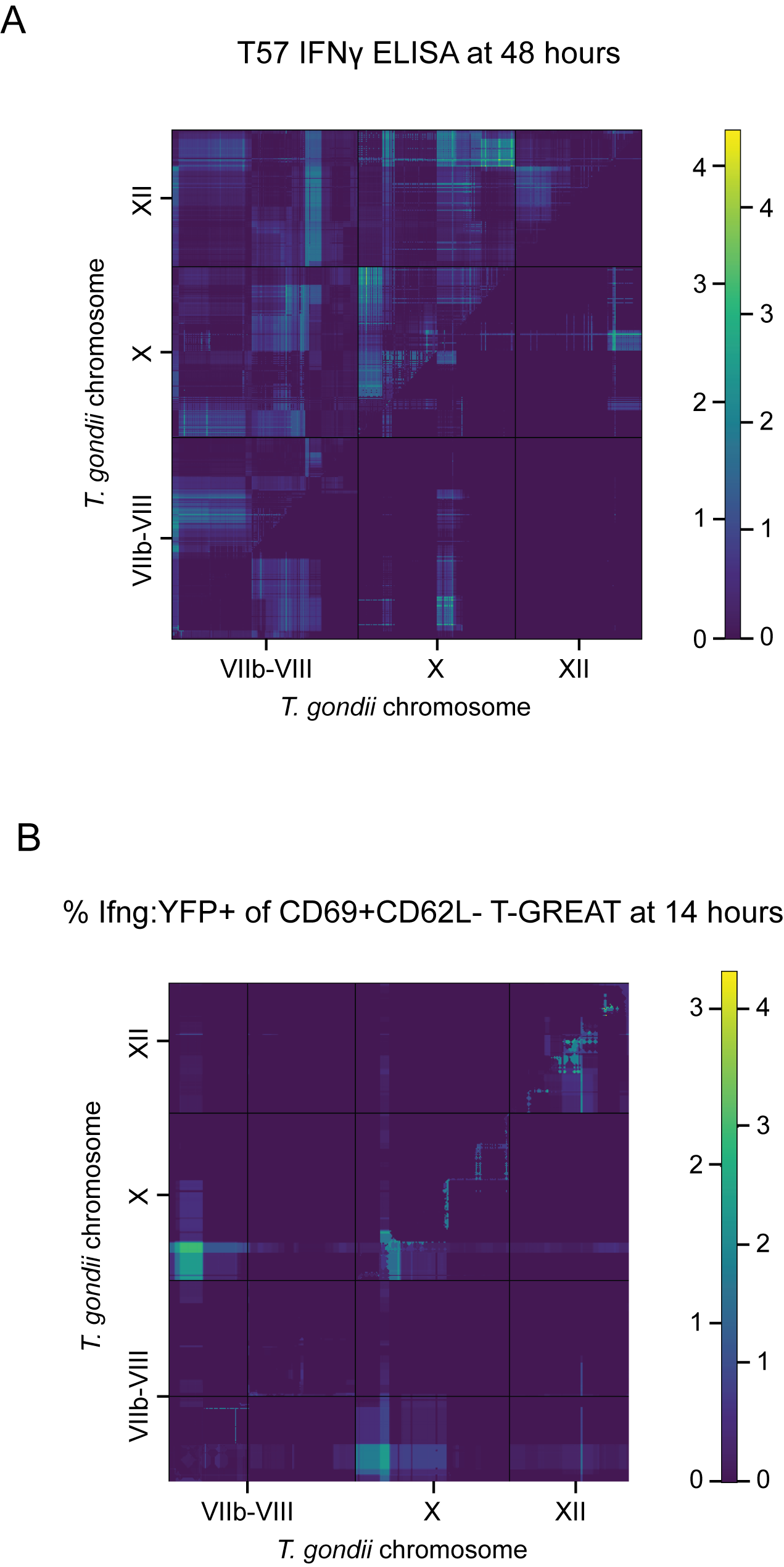

Supplement: Supplementary Figure 2 — A two-dimensional (2D) QTL genome-wide scan does not detect interactive effects between loci associated with CD8 T cell IFNγ responses to T. gondii infections. (A) 2D QTL analysis of T57 IFNγ secretion at 48 hours to T. gondii F1 IxII infections. The selected T. gondii chromosomes are labeled on both axes. The LOD scores in the 2D plot are represented on a color scale indicated on the right. The LOD score scale from 0 to 4.2 corresponds to the upper left triangle which compares the full two-locus or “interactive QTL” model, in which all possible combinations of two markers are calculated for epistasis, to an additive-QTL model. The LOD score scale from 0 to 4.8 corresponds to the lower right triangle of the 2D plot, which compares the two-locus to a single-locus QTL model. No two pairs of loci in the two-locus model surpass a p-value of p = 0.1 (LOD > 8), and no advantage is gained over a single-QTL or additive-QTL model (R/qtl). (B) As in (A), but 2D QTL analysis of the Ifng : YFP+ frequency of CD69+ CD62L- T-GREAT T cells at 14 hours of co-culture with F1 IxII infectioned BMDMs is plotted. No two pairs of loci in the two-locus model surpass a p-value of p = 0.1, and no advantage is gained over a single-QTL or additive-QTL model (R/qtl). [file Image_2.tif]

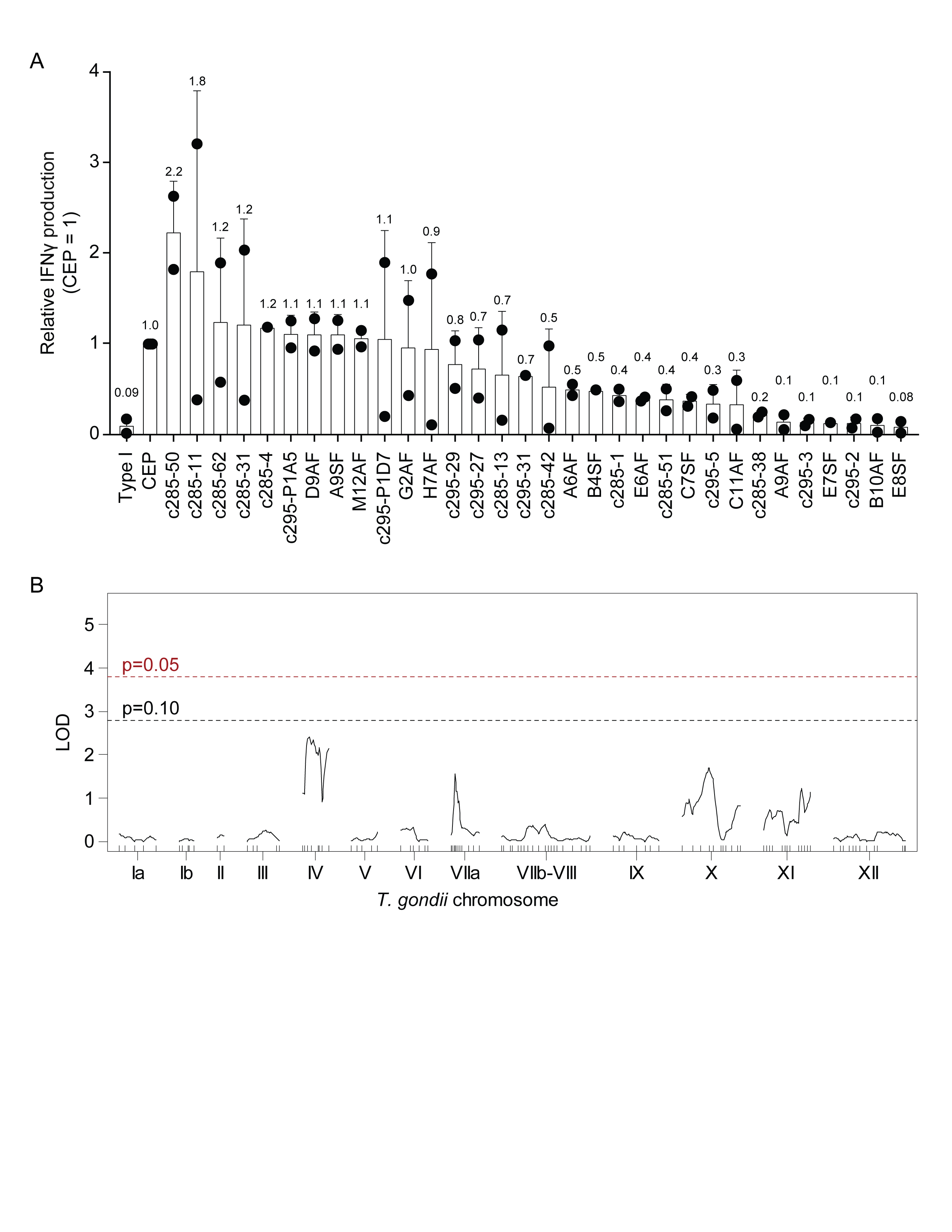

Supplement: Supplementary Figure 3 — Genetic linkage analysis of TGD057-specific CD8 T cell IFNγ responses to Toxoplasma gondii F1 progeny of the type I x III cross reveals no significant QTLs. (A) T57 CD8 T cell IFNγ responses to BMDM infections with F1 IxIII T. gondii strains were measured as previously described in Figure 1 and normalized to that induced by the type III CEP strain. The normalized values are indicated above the bar graphs. Average of 2 experiments +SD are shown, each dot represents the results from one experiment. (B) A genome-wide QTL scan of the CD8 T cell IFNγ response to F1 IxIII T. gondii-infected BMDMs was performed. The running LOD score for each genetic marker is shown; T. gondii chromosomes are indicated. Significant threshold LOD values of p = 0.05 and p = 0.10 following 1,000 permutations are indicated in red and black, respectively. [file Image_3.tif]

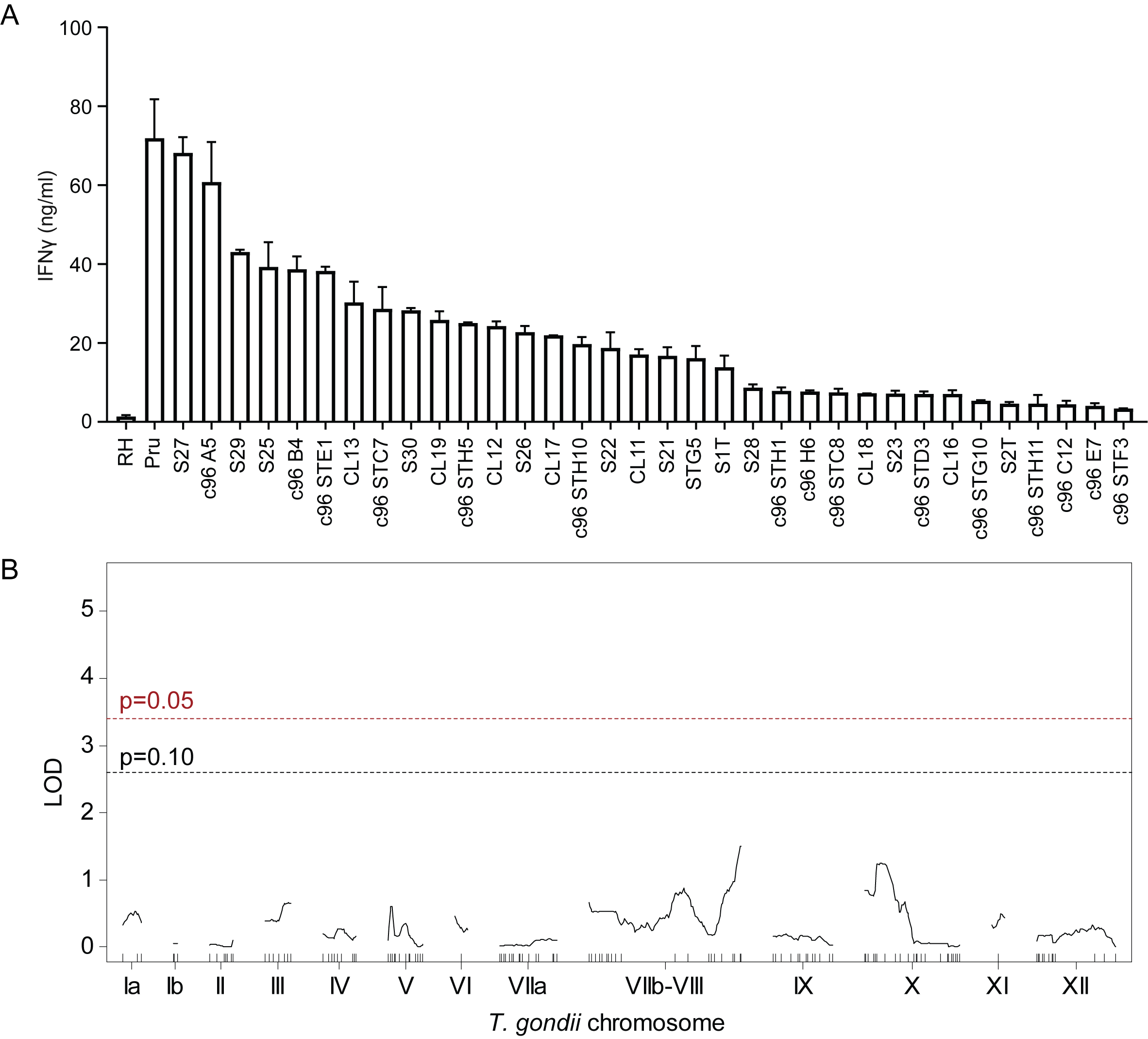

Supplement: Supplementary Figure 4 — Genetic linkage analysis of TGD057-specific CD8 T cell IFNγ responses to Toxoplasma gondii F1 progeny of the type II x III cross reveals no significant QTLs. (A) F1 IIxIII T. gondii strains were assayed for T57 CD8 T cell IFNγ responses as described in Figure 1 . Plotted is the average IFNγ concentration in the supernatant at 48h post addition of T57 CD8 T cells +SD of 3 technical replicates from a single experiment. (B) A genome-wide QTL scan of the CD8 T cell IFNγ response to F1 IIxIII T. gondii BMDM infections was performed and the running LOD score for each T. gondii genetic marker is shown. The significant threshold LOD values of p = 0.05 and p = 0.10 following 1,000 permutations are indicated in red and black, respectively. [file Image_4.tif]

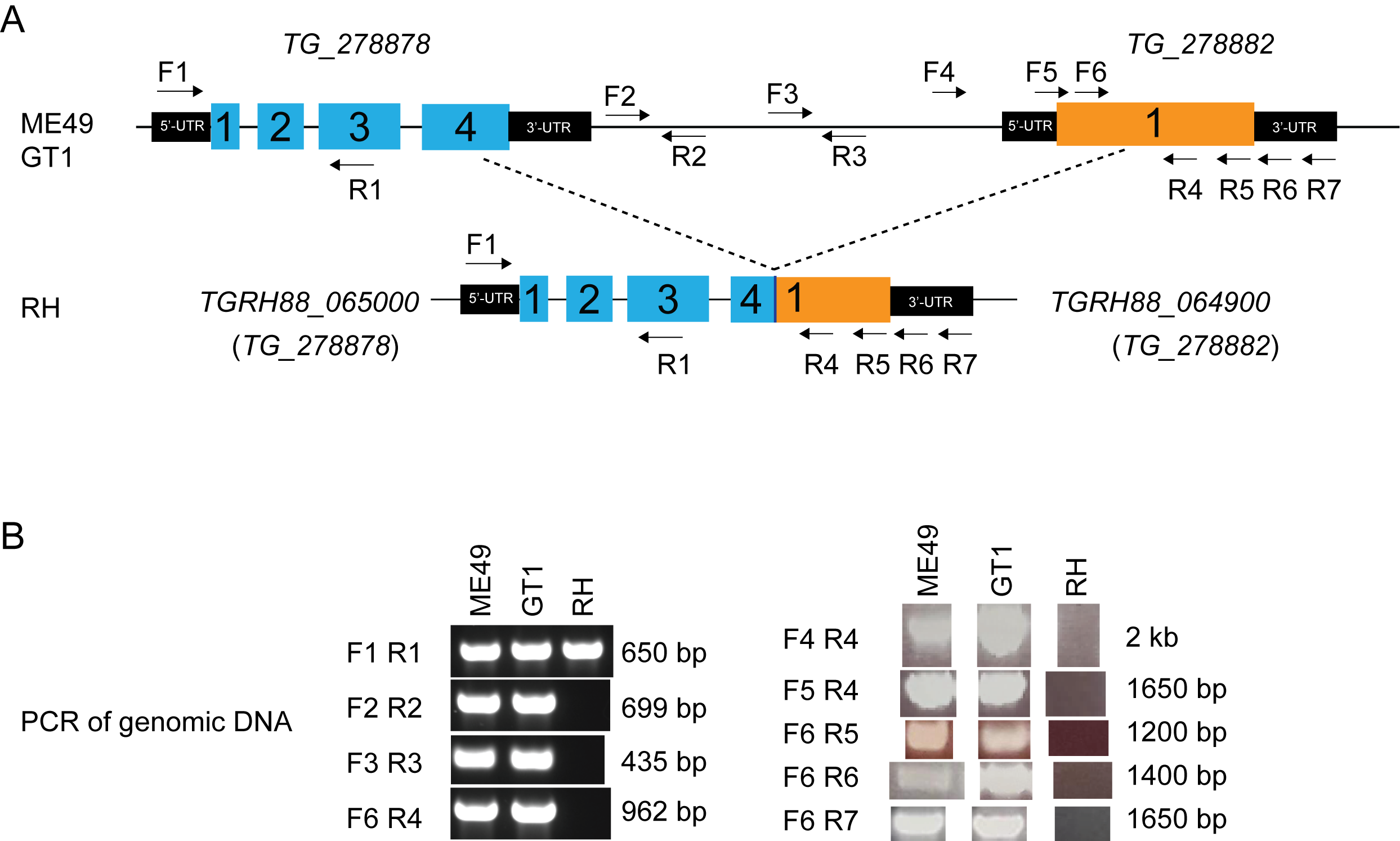

Supplement: Supplementary Figure 5 — A large-scale deletion occurred in the NTPase locus of the RH genetic background. (A) Schematic of the NTPase locus encoding the genes TG_278878 and TG_278882 in the ME49 (type II), GT1 and RH (type I) genetic backgrounds. TGRH88_065000 and TGRH88_064900 are syntenic to TG_278878 and TG_278882, respectively. The RH genome (GCA_013099955.1) reveals a 4.5 Kb deletion occurred at this locus, in which the 3’ end of exon 4 for TG_278878 experienced a 338 bp truncation and was then fused to a TG_278882 gene missing 1.2 Kb of the 5’ end of exon 1. The fusion event is indicated by dashed lines. (B) PCR results obtained with the indicated primers in (A), confirms this deletion within RH Δku80 Δhxgprt, but not GT1 and ME49 Δhxgprt parasite strains. [file Image_5.tif]

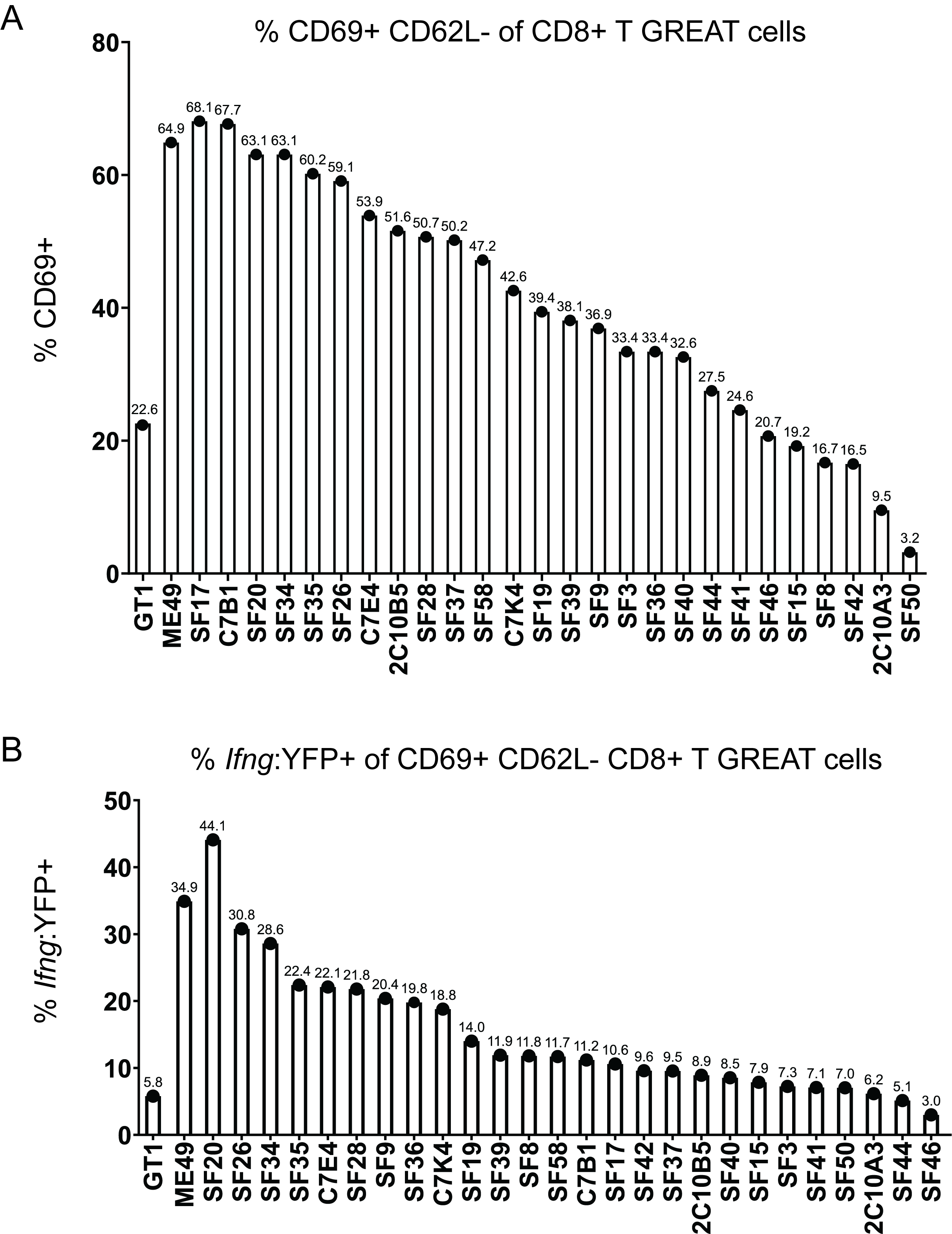

Supplement: Supplementary Figure 6 — CD69 and Ifng expression profiles of T-GREAT CD8 T cells in response to individual Toxoplasma gondii F1 progeny of the type I x type II cross. (A) TGD057-specific T-GREAT CD8+ T cell responses to T. gondii-infected BMDMs were assayed at 14 hours, as described in Figure 6 , with F1 progeny of the type I x II cross (F1 IxII). Frequency of activated CD69+ CD62L- cells among total CD3+ CD8+ T-GREAT cells is plotted. (B) As in (A), but the frequency of Ifng : YFP+ cells among total CD69+ CD62L- CD3+ CD8+ T-GREAT are shown. Results are from an individual experiment; values are indicated above the bar graphs. [file Image_6.tif]
